# Spatiotemporally resolved transcriptomics reveals the subcellular RNA kinetic landscape

**DOI:** 10.1038/s41592-023-01829-8

**Published:** 2023-04-10

**Authors:** Jingyi Ren, Haowen Zhou, Hu Zeng, Connie Kangni Wang, Jiahao Huang, Xiaojie Qiu, Xin Sui, Qiang Li, Xunwei Wu, Zuwan Lin, Jennifer A. Lo, Kamal Maher, Yichun He, Xin Tang, Judson Lam, Hongyu Chen, Brian Li, David E. Fisher, Jia Liu, Xiao Wang

**Affiliations:** 1 Department of Chemistry, Massachusetts Institute of Technology, Cambridge, MA USA; 2 Broad Institute of MIT and Harvard, Cambridge, MA USA; 3 Whitehead Institute for Biomedical Research Cambridge, Cambridge, MA USA; 4 John A. Paulson School of Engineering and Applied Sciences, Harvard University, Boston, MA USA; 5 Cutaneous Biology Research Center, Massachusetts General Hospital, Harvard Medical School, Charlestown, MA USA; 6 Department of Chemistry and Chemical Biology, Harvard University, Cambridge, MA USA; 7 Computational and Systems Biology Program, Massachusetts Institute of Technology, Cambridge, MA USA

**Keywords:** Transcriptomics, RNA sequencing, RNA metabolism, Molecular biophysics, RNA

## Abstract

Spatiotemporal regulation of the cellular transcriptome is crucial for proper protein expression and cellular function. However, the intricate subcellular dynamics of RNA remain obscured due to the limitations of existing transcriptomics methods. Here, we report TEMPOmap—a method that uncovers subcellular RNA profiles across time and space at the single-cell level. TEMPOmap integrates pulse-chase metabolic labeling with highly multiplexed three-dimensional in situ sequencing to simultaneously profile the age and location of individual RNA molecules. Using TEMPOmap, we constructed the subcellular RNA kinetic landscape in various human cells from transcription and translocation to degradation. Clustering analysis of RNA kinetic parameters across single cells revealed ‘kinetic gene clusters’ whose expression patterns were shaped by multistep kinetic sculpting. Importantly, these kinetic gene clusters are functionally segregated, suggesting that subcellular RNA kinetics are differentially regulated in a cell-state- and cell-type-dependent manner. Spatiotemporally resolved transcriptomics provides a gateway to uncovering new spatiotemporal gene regulation principles.

## Abstract (web summary)

TEMPOmap combines pulse-chase metabolic labeling with multiplexed three-dimensional in situ sequencing to simultaneously profile the age and subcellular location of individual RNA molecules from thousands of genes to reveal RNA kinetic landscapes.

## Main

Cell state and function are shaped by the spatiotemporal regulation of gene expression. This heterogeneous expression is achieved, in part, through precise mRNA metabolism and trafficking over time. The ability to systematically profile transcriptomes across time and space at a single-cell level from intact cellular networks is critical to understanding transcriptional and posttranscriptional gene regulatory mechanisms in cells and tissues.

However, current transcriptomic approaches are unable to simultaneously capture both the spatial and time dependence of RNA profiles. For instance, spatially resolved transcriptomics methods have enabled integrated profiling of gene expression from heterogeneous cell types in the context of tissue morphology^1–7^. Nonetheless, these spatial transcriptomics approaches alone can provide only static snapshots of cells and tissues, while the dynamic flow of gene expression cannot be determined^8^. In contrast, existing metabolic RNA labeling approaches have enabled temporal profiling of the nascent single-cell transcriptome but lack spatial resolution^9–13^. In addition, live-cell imaging can directly track RNA trajectory inside cells, but simultaneously visualizing multiplexed transcripts remains challenging^14^. Thus, there exists a pressing need for highly multiplexed, spatially and temporally resolved sequencing methods that track nascent mRNAs in situ from birth to death at subcellular and single-cell resolutions.

**Fig. 1:**
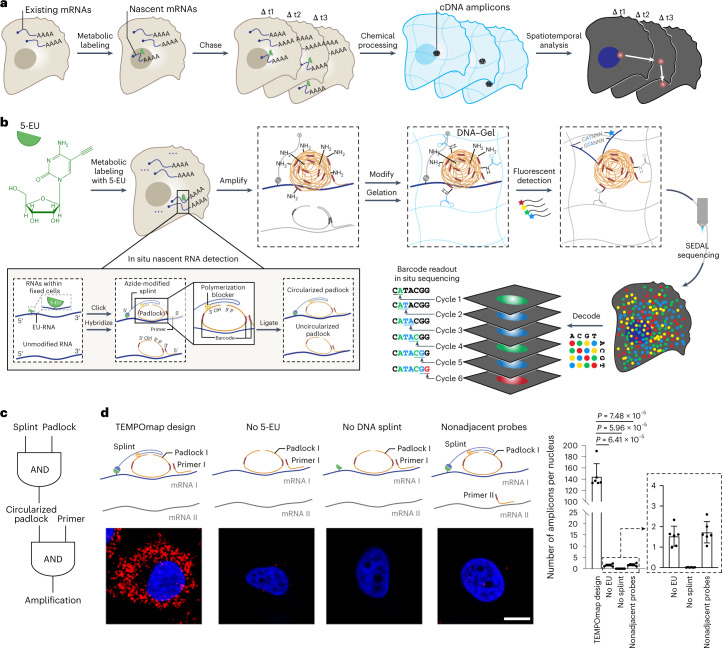
TEMPOmap enables spatiotemporally resolved transcriptomics. **a**, Overview of TEMPOmap pipeline: nascent RNAs of several timepoints are collected and in situ sequenced, followed by spatiotemporal RNA analyses. **b**, TEMPOmap experimental workflow. After 5-EU-labeled cells are prepared, a set of tri-probes (splint, primer and padlock) are conjugated or hybridized to cellular mRNAs (Extended Data Fig. 1b,i for more details), resulting in the enzymatic replication of each padlock sequence into cDNA amplicons. The amplicons are anchored in situ via a functionalized acrylic group (blue) to a hydrogel mesh to create a DNA-gel hybrid (blue wavy lines). The five-base barcode on each amplicon is read out by six rounds of SEDAL. Thus, multiplexed RNA quantification reveals gene expression in nascent subcellular locations. **c**, DNA tri-probe design rationale. The generation of an amplicon requires the presence of splint, circularized padlock and primer probes in proximity. **d**, Left, schematics and representative fluorescent cell images of negative control experiments of **c**, showing three-part probe requirement for signal amplification. mRNA_I represents ACTB and mRNA_II represents GAPDH. All four images show ACTB (red) mRNA in HeLa cells (DAPI in blue). Right, quantification of cell images showing the average amplicon reads per cell (*n* = 6 images were measured containing 310, 585, 386 and 421 cells for each condition from left to right, respectively). Two-tailed *t*-test. Data shown as mean ± s.d. Scale bar, 10 µm.

**Extended Data Fig. 1:**
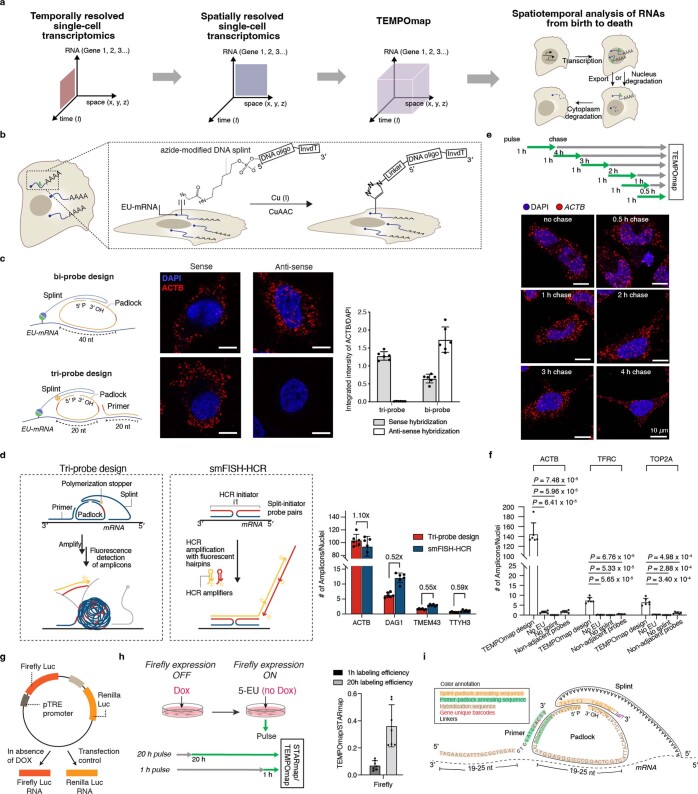
TEMPOmap experimental design and optimization. **a**, Method conceptualization. TEMPOmap combines RNA metabolic labeling and state-of-the-art spatial transcriptomics to achieve single-cell spatiotemporal transcriptomics for RNA dynamic analysis. **b**, CuAAC-mediated click chemistry to conjugate azide-modified splint and EU-labeled nascent transcript. **c**, Comparison of TEMPOmap bi-probe and tri-probe design targeting *ACTB* mRNA. Left, probe design schematics. Middle, representative fluorescent images of cells treated with sense-targeting and antisense-targeting padlocks and primers. Right, quantification of fluorescence in cell images (6 images containing 400-600 cells were measured under each condition). Data shown as mean + s.d. **d**, left: schematics showing the probe design and experiment of tri-probe and smFISH-HCR. Tri-probe consists of three probes (primer, padlock, and splint) that target the same region of RNA targets, where the circularized padlock will be amplified to generate amplicons when all three probes are hybridizing on the same RNA locus. smFISH-HCR probe sets target to the same RNA regions, and bound probe pairs trigger self-assembly of a tethered fluorescent amplification polymer (or amplicon); right: quantification of the averaged number of amplicons per cell generated from tri-probe design or smFISH-HCR when targeting four genes with different expression levels (*ACTB, DAG1, TMEM43, TTYH3*) (n = 6 images per condition). **e**, Proof-of-concept pulse-chase experiment (top) followed by raw cell images (bottom) showing the translocation of *ACTB* mRNAs when chased after 1 hr EU treatment with different times. Cell nuclei (blue), amplicons (red). **f**, quantification of the averaged amplicon reads per cell (n = 6 images for each experimental condition). Unpaired two-sided t-test. **g**, schematics showing the mechanism of action of DNA vector expressing Firefly luciferase (under the control of DOX-induced promoter) and Renilla luciferase (under the control of constitutive promoter). **h**, left: schematics showing the two EU pulse-labeling timepoints while inducing the expression of Firefly luciferase for the same duration of time, followed by TEMPOmap/STARmap experiment; right: the quantification of the ratios of the average amplicon counts by TEMPOmap over STARmap for each field of view (n = 7 images). **i**, DNA sequences of TEMPOmap tri-probe system. Data shown as mean + s.d. Scale bar: 10 µm.

Here, to provide a systematic single-cell analysis of RNA life cycle in time and space, we introduce TEMPOmap (temporally resolved in situ sequencing and mapping)—a method that tracks the spatiotemporal evolution of the nascent transcriptomes over time at subcellular resolution (Extended Data Fig. 1a). TEMPOmap integrates metabolic labeling and selective amplification of pulse-labeled nascent transcriptomes with the current state-of-the-art three-dimensional (3D) in situ RNA sequencing at 200 nm resolution within a hydrogel-cell scaffold^1^ (Fig. 1a). Using pulse-chase labeling, we were able to simultaneously track key kinetic parameters for hundreds to thousands of genes during their RNA life cycle, including rates of transcription, decay, nuclear export and cytoplasmic translocation. Using these spatiotemporal parameters, we show that mRNAs of different genes are differentially regulated at different steps of the RNA life cycle and across different cell-cycle phases, which ultimately serves gene functions.

## Results

### TEMPOmap for spatiotemporally resolved transcriptomics

**Extended Data Fig. 2:**
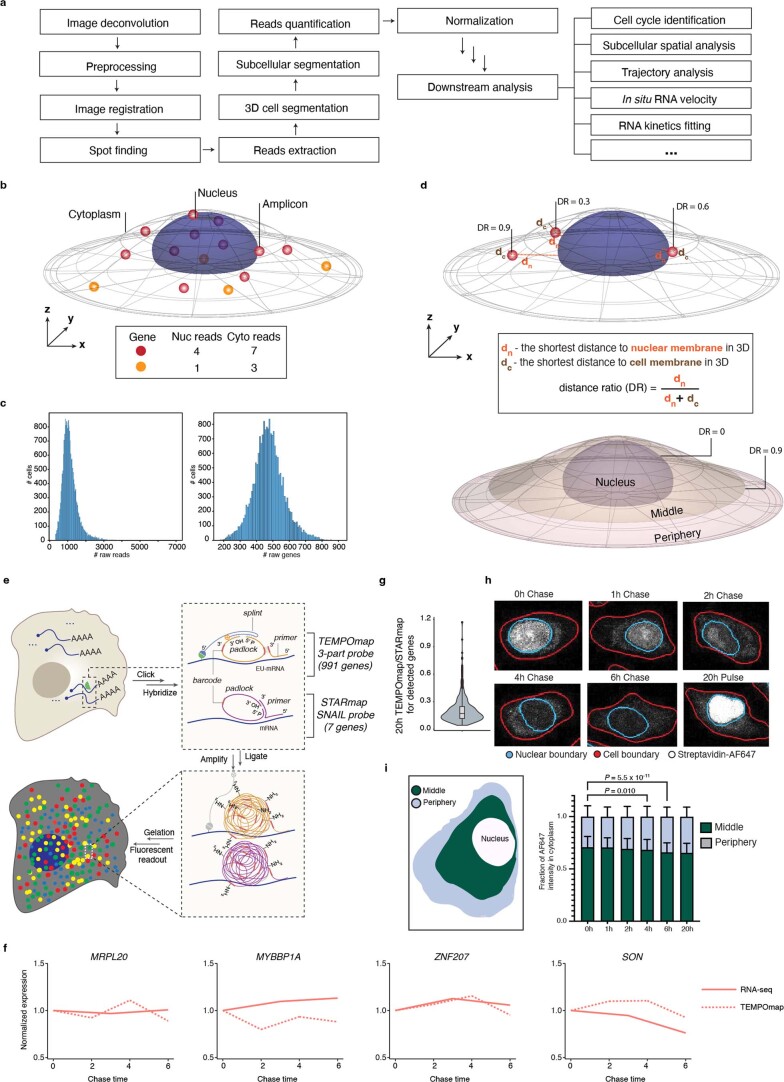
TEMPOmap data processing and analysis. **a**, TEMPOmap data analysis pipeline. **b**, Schematics of reads assignment in subcellular compartments. **c**, Histograms showing detected reads (cDNA amplicons) per cell (left), and genes per cell (right). **d**, Schematics of distance ratio (DR)-based subcellular segmentation in the cytoplasm. Two values for each amplicon were computed in 3D: d_n_, the shortest distance to nuclear membrane; d_c_, the shortest distance to cell membrane. ‘Middle’ is the region defined between DR = 0 and 0.9. ‘Periphery’ is defined as DR > 0.9. **e**, Simultaneous mapping and sequencing of nascent RNAs by TEMPOmap and total RNAs by STARmap in the experimental workflow. TEMPOmap-targeted amplicon reads were normalized against the reads of STARmap-targeted RNAs. **f**, validation of normalization strategy by comparing the gene expression data of example RNAs between TEMPOmap and bulk RNA-seq^34^. Both datasets were normalized by the first timepoint to facilitate the comparison of change in gene expression. **g**, violin plot showing the distribution of the ratios of TEMPOmap reads over STARmap reads averaged across single cells for all detected genes (n = 991 genes). Boxplots are defined in terms of mean (center line), 25-75% percentile (bounds of box), lower and upper quartile (whiskers) and outlier values (dots). **h**, representative fluorescent images showing the subcellular distribution of streptavidin fluorescence across pulse-chase labeling times. **i**, left: subcellular region assignment (middle and periphery) of one representative cell; right: boxplot summarizing the fraction of reads in two subcellular regions of single cells at each timepoint. Data are presented as mean values + /- s.d. The statistics compare the fractions of reads in the periphery. Kruskal–Wallis test with post hoc Tukey’s HSD. n = 238, 310, 171, 314, 333, 286 cells in each timepoint from left to right.

TEMPOmap begins with metabolically labeling cultured cells with 5-ethynyl uridine (5-EU)^13,15^, which adds a bioorthogonal chemical handle on the labeled mRNAs (Fig. 1b). Next, we designed a tri-probe set (splint, padlock and primer) for each mRNA species to selectively generate complementary DNA amplicons derived from metabolically labeled RNAs (Fig. 1b,c): (1) the splint DNA probe contains 5′ azide- and 3′ chain-terminator groups to be covalently attached with the 5-EU-labeled mRNAs via copper(I)-catalyzed azide-alkyne cycloaddition (CuAAC; Extended Data Fig. 1b), thus excluding unlabeled RNAs from subsequent cDNA amplification; (2) the padlock probes recognize mRNA targets with a complementary 20–25 nucleotide cDNA sequence, contain gene barcodes and can be circularized when the splint probe is in physical proximity on the same RNA; (3) the primer probes target the neighboring 20–25 nucleotides next to the padlock probes, which serve as the primer to amplify circularized padlocks in situ via rolling cycle amplification (RCA), forming cDNA nanoballs (amplicons); in combination, only mRNAs that are binding simultaneously to all three types of probes will be amplified, therefore allowing selective detection of labeled mRNA population in a label- and sequence-specific manner via a two-step thresholding strategy (Fig. 1d). Notably, a single gene-targeting padlock probe (bi-probe design) cannot achieve specific gene detection (Extended Data Fig. 1c), confirming the necessity of the dual gene-targeting primer and padlock pair in the tri-probe design^1^. When all three components of the tri-probe are present at the same target transcript, we estimated the detection efficiency to be around 50–100% of smFISH-HCR (Extended Data Fig. 1d). As a proof of concept, we tested representative tri-probes targeted genes with high expression and medium-to-low expression in HeLa cells. We found that 5-EU labeling and all three probes were necessary for signal amplification, demonstrating specific detection of metabolically labeled transcripts (Fig. 1d and Extended Data Fig. 1e,f). By targeting chemically induced (firefly luciferase) reporter gene (Methods), we further estimated the detection efficiency of TEMPOmap tri-probe on EU-labeled transcripts to be around 36% for 20 h EU incubation, and 7% for 1 h EU incubation (Extended Data Fig. 1g,h). To enable highly multiplexed transcriptome detection, the in situ generated cDNA amplicon libraries are subsequently embedded in a hydrogel matrix for several cycles of fluorescent imaging to decode the gene-encoding barcodes via SEDAL (sequencing with error-reduction by dynamic annealing and ligation) (Fig. 1b and Extended Data Fig. 1i) to simultaneously profile hundreds to thousands of genes. After the completion of sequencing cycles, the amplicon reads are registered, decoded and subjected to 3D segmentation for subcellular and single-cell resolved analysis (Extended Data Fig. 2a).

### Spatiotemporal evolution of single-cell transcriptome

**Fig. 2:**
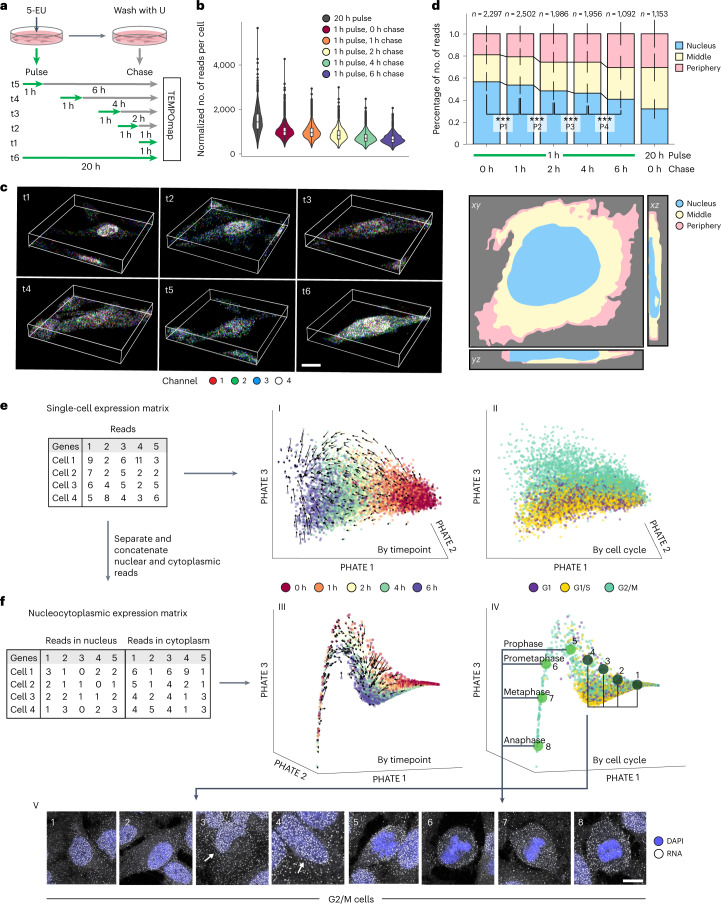
Spatiotemporal tracing of single-cell transcriptome. **a**, Pulse-chase experiment design on HeLa cells. For the first five timepoints, we used 1 h metabolic labeling (pulse) followed by 0, 1, 2, 4 and 6 h chase. At the last timepoint, we labeled the cells metabolically for 20 h. All cells were then processed by TEMPOmap workflow measuring 998 genes. **b**, RNA reads (cDNA amplicons) per cell for each pulse-chase timepoint. *n* = 1,425; 4,024; 4,421; 3,521; 3,727 and 2,303 cells from different pulse-chase timepoints (left to right). Boxplots are defined in terms of mean (center line), 25–75% percentile (bounds of box), lower and upper quartile (whiskers) and outlier values (dots). **c**, 3D fluorescent images of inprocess TEMPOmap with zoomed-in views of representative single cells in sequencing cycle 1 at each timepoint. Z-stack range, 10 µm. Scale bar, 10 µm. **d**, Top, stacked bar plot summarizing the fraction of reads in each subcellular region of all cells at each timepoint. Data are presented as mean values ± s.d. The statistics compare the fractions of nuclear reads (blue) across the first five timepoints. ***P < 0.001, Kruskal–Wallis test with post hoc Tukey’s honestly significant difference test. The number of cells (*n*) at each timepoint is shown. Bottom, subcellular region assignment (nuclear, middle and periphery) of one representative cell. **e**–**f**, TEMPOmap single-cell (**e**) or nucleocytoplasmic (**f**) RNA measurements rendered as a visualization by PHATE and colored by pulse-chase timepoints (I, III) or cell-cycle marker gene expression (II, IV). Black arrows inferred by RNA degradation vectors indicate the directions of chase time progression. Bottom row, representative raw images of G2/M phase cells separated on PHATE coordinates. All images show mRNAs (white) in HeLa cells (DAPI in blue). Scale bar, 15 µm.

**Extended Data Fig. 3:**
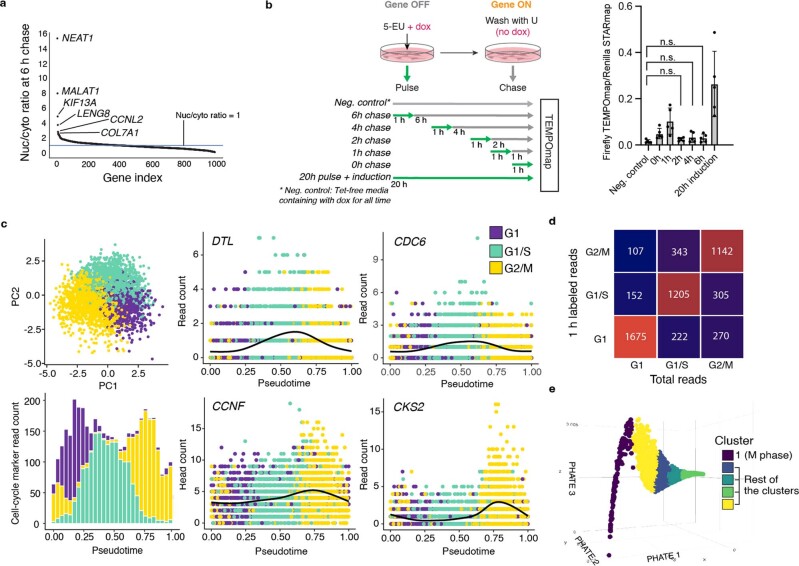
RNA subcellular analysis, residual EUTP test and cell-cycle phase identification. **a**, nuclear-to-cytoplasmic ratio of amplicon reads of 991 genes at 6 hrs chase timepoint. Genes were ranked from top to bottom according to the ratios. **b**, left: pulse-chase experiment design on HeLa tet-OFF cells. We used 1 hr metabolic labeling (pulse) followed by 0, 1, 2, 4, and 6 hrs chase while inducing the gene expression of Firefly luciferase. We added a negative control with no gene expression and no labeling, and a positive control with 20 h pulse and gene induction. All of the cells were then processed by TEMPOmap workflow. Right: quantification of the averaged number of amplicons of Firefly TEMPOmap normalized by Renilla STARmap in each condition. n = 5 fluorescent images measured per condition. Data are presented as mean values + /- s.d. n.s = non-significant, two-tailed *t-*test. **c**, Cell-cycle identification (G1, G1/S, G2/M) by cell-cycle gene marker measured via TEMPOmap labeled RNA expression. Cell-cycle scores were calculated via ‘score_genes_cell_cycle‘ in scanpy. The cells were visualized via PCA and colored by cell-cycle phases (top left). Variations in the raw counts of all cell-cycle gene markers (bottom left) and four representative markers (right) were projected by the pseudotime analysis. **d**, Comparison of cell-cycle identification by 1 hr pulse-labeled reads and total reads using scEU-seq dataset^13^ shows that the nascent transcriptome can accurately define cell-cycle states. The number in each box indicates the number of cells. **e**, Cell clustering result based on PHATE embedding of the nucleocytoplasmic matrix. Cluster 1 incorporates the cells (*n* = 137 cells) in the M phase by visually inspecting raw images.

To assess TEMPOmap in human cells, we mapped a curated list of 991 genes (981 coding, 10 noncoding RNA) with diversified spatial and temporal RNA expression profiles^13,16^ in HeLa cell cultures (Supplementary Table 1). Then, we designed a pulse-chase experiment with 1 h pulse labeling and various chase times (0, 1, 2, 4 and 6 h) as well as one steady-state reference with 20-h pulse labeling (Fig. 2a), followed by the TEMPOmap experiment workflow (Fig. 1b). The barcodes in all the samples were sequenced over six rounds of in situ sequencing, followed by a final round of subcellular compartment staining (nuclei and cytoplasm) to segment cell bodies and assign the subcellular locations of amplicons in 19,856 cells in 3D (Extended Data Fig. 2b–d; Methods). Notably, we conducted normalization using internal reference genes (STARmap-targeting genes; Extended Data Fig. 2e,f) for batch correction to minimize potential technical variation across timepoints. The detection efficiency of TEMPOmap across all genes is around 20.5% estimated by pairwise 20-h labeling and STARmap (Extended Data Fig. 2g). Importantly, from 0 to 6 h chase time postlabeling, we observed a decline of total RNA reads per cell, a gradual shift of the RNA distribution from the nucleus to the cytoplasm and further allocation from the middle cytoplasmic region to the periphery (Fig. 2c,d), in agreement with the expected trajectory of RNAs (Extended Data Fig. 2h,i). Interestingly, a substantial fraction of reads (around 40%) was retained in the nucleus even after 6 h. A closer inspection of the retained RNA molecules revealed that RNAs with the highest nuclear-to-cytoplasm read ratio included long noncoding RNAs (*NEAT1*, *MALAT1*), supported by deep sequencing of RNA from cellular fractions (Extended Data Fig. 3a)^17,18^. Notably, a group of mRNAs (for example, *KIF13A*, *LENG8*, *CCNL2*, *COL7A*) showed high ratios of nuclear retention (nuc:cyto >2; Extended Data Fig. 3a). To rule out the possibility that the observed nuclear retention was an artifact of nascent transcript labeling with residual ethynyl-UTP (EUTP) during the chase, we utilized the aforementioned inducible firefly luciferase construct. After inducing firefly luciferase mRNA transcription by removing EU-containing medium, the EUTP incorporation was minimal after 1–2 h as detected by TEMPOmap probes against firefly luciferase, suggesting a temporal resolution of pulsed labeling of 1–2 h (Extended Data Fig. 3b). Our observations validate the previous discovery of nuclear retention of mRNA, which may serve as a regulatory role to buffer cytoplasmic gene expression noise^19,20^.

Next, we asked whether the TEMPOmap dataset could resolve the heterogeneity of RNA posttranscriptional dynamics in single cells. To this end, we pooled all the cells under the 1 h pulse conditions (18,176 cells) for single-cell resolved dynamic trajectory analysis using potential of heat-diffusion for affinity-based trajectory embedding (PHATE) (Fig. 2e,I)^21,22^. Our results showed a clear trajectory along the progression of chase time, which results from differential RNA degradation rates among genes and suggests that the temporally resolved single-cell transcriptional states could be distinguished and aligned readily in the latent space. Overlaying the same coordinates with RNA degradation kinetics vectors (represented as the quivers) further recapitulated the single-cell trajectory along RNA life cycle progression^22–24^ (Methods). We then investigated how the RNA life cycle defined by the pulse-chase timeline aligns with cell-cycle progression. To this end, we classified the cells into three cell-cycle phases (G1, G1/S and G2/M) based on their nascent expression of marker genes (Extended Data Fig. 3c,d) using cell-cycle scoring^25^ (Methods). Interestingly, the direction of cell-cycle progression is orthogonal to that of the pulse-chase timepoint progression (Fig. 2e,II). This observation suggests that TEMPOmap provided independent temporal information regarding the RNA life cycle in addition to the cell cycle.

Beyond single-cell analysis, we considered that the TEMPOmap dataset could reveal subcellular RNA dynamics. To this end, we generated a nucleocytoplasmic gene-by-cell matrix by concatenating single-cell nuclear expression with cytoplasmic expression for trajectory analysis (Fig. 2f). Apart from recovering the unidirectional trajectory of single cells along with the labeling timepoints (timepoint III; Fig. 2f), we found a small fraction (*n* = 137 cells; 2.1%) of G2/M cells that formed a narrow trajectory and projected into a distinct space, suggesting that the nucleocytoplasmic RNA distribution in this group of G2/M cells differs drastically from the rest of the G2/M cells (Extended Data Fig. 3e). We suspected that these spatially distinct cells were the cells undergoing mitosis with their unique RNA nucleocytoplasmic distribution^26^. Indeed, the cells on this trajectory had been in different phases of mitosis, during which RNAs were mostly evicted from the chromatin regions compared with that in G2 cells (Fig. 2f,V). Furthermore, the uniform direction of this distinct trajectory aligns well with the time progression of mitosis (Fig. 2f, V, 5–8), indicating that the temporal mitotic transitions could be inferred by subcellular RNA localization patterns. As a result, by jointly making use of the time-gated nucleocytoplasmic distribution, we not only separated G2 and M cells but also traced the trajectory of mitosis on the gene expression space, during which there is drastic RNA eviction from chromosomes in M cells^27^.

### Subcellular RNA kinetic landscape across RNA lifespan

**Fig. 3:**
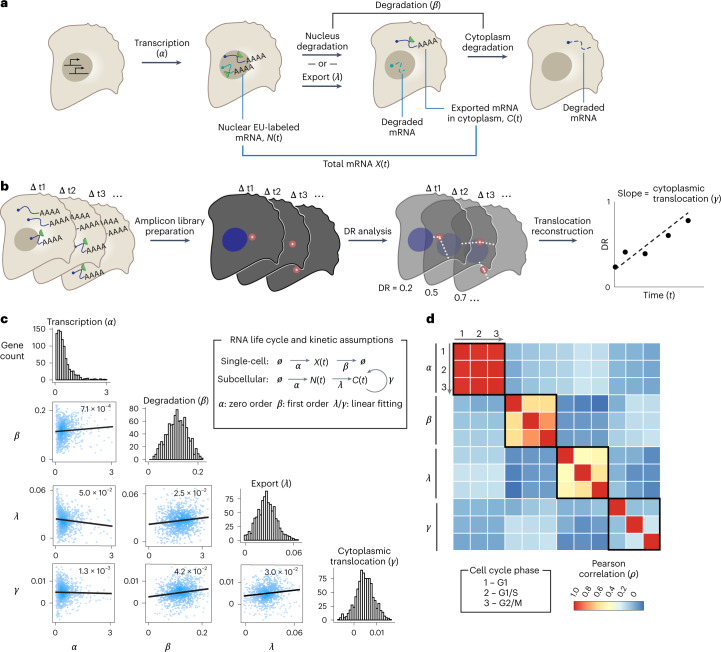
TEMPOmap reveals subcellular RNA kinetic landscape across RNA lifespan and cell cycle. **a**, Dynamic model for estimating RNA kinetic parameters. For each gene, RNA synthesis (*α*) and degradation constant (*β*) were estimated using single-cell RNA concentration. The export constant (*λ*) was estimated using the subcellular RNA reads. **b**, The dynamic model for estimating cytoplasmic translocation (*γ*) using DR-based analysis (Methods). **c**, Upper right, mathematical model of RNA life cycle and kinetic assumptions used for the parameter estimation. Bottom left, histogram of the four parameters for all genes that passed qualify control and scatterplots depicting the pairwise correlation of parameters with *r* value (Pearson correlation) and linear fitting curve. Color intensity of the dots indicates local density. Units of *α* is RNA concentration per hour, where RNA concentration is defined by copies per voxel (voxel = 200 nm × 200 nm × 350 nm). Units of *β*, *λ*, *γ* are per hour. **d**, Heatmap depicting pairwise correlation matrix of the four parameters estimated using single cells from three cell-cycle phases (G1, G1/S, G2/M). Color indicates the value of Pearson correlation coefficients. Boxed regions indicate the correlations of each parameter among three cell-cycle phases.

**Extended Data Fig. 4:**
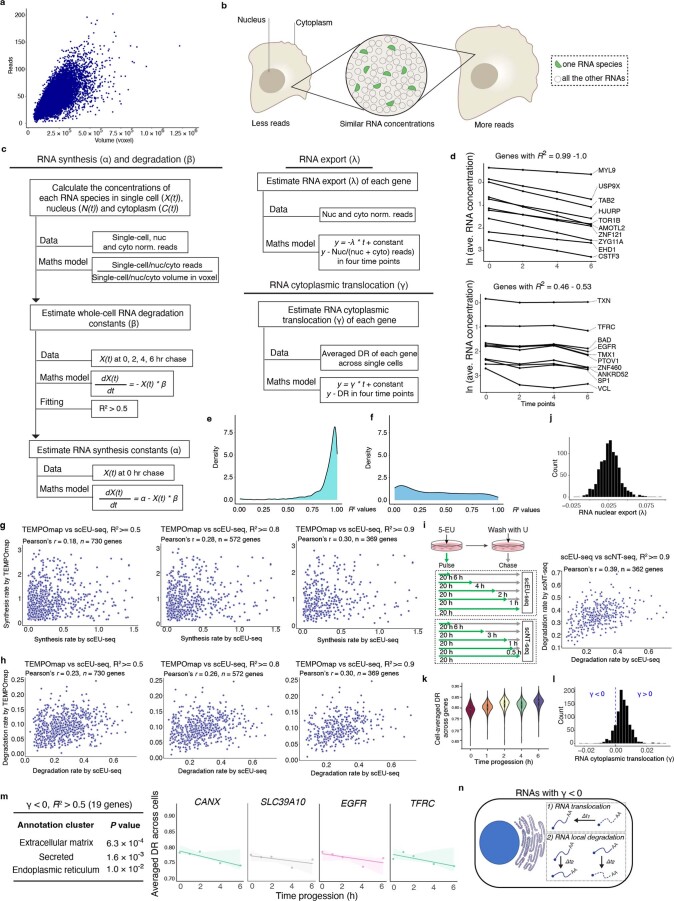
Quantification and quality control of RNA subcellular kinetic parameters. **a**, Correlated relation between cell volume (in voxels) and single-cell reads, indicating the influence of transcript number by cell volume. **b**, Schematics showing the biased single-cell RNA read counts with varying physical cell volumes. **c**, Mathematical models for estimating RNA kinetic parameters (α,β and λ) and the detailed workflow of calculation and fitting procedure. Note: *X(t)* = single-cell RNA concentration; *N(t)* = nuclear RNA concentration; *C(t)* = cytoplasmic RNA concentration. **d**, Changes in the natural log of *X(t)* across timepoints of genes with R^2^ = 0.99-1.0 (top) and R^2^ = 0.46-0.53 (down). Representative genes are shown. The estimated β values for all genes were filtered with a threshold of R^2^ > 0.5 as quality control. **e-f**, Density plots showing the distribution of R^2^ when fitting degradation constant by a first-order ODE using original (**e**) and temporally-shuffled (**f**) pseudobulk expression vectors. **g-h**, scatterplots showing Pearson’s correlation of RNA synthesis (RNA/hour, **g**) and degradation (1/hr, **h**) estimation between this study and single-cell scEU-seq datasets after filtering the genes based on fitting the degradation parameters using R^2^ > = 0.5, 0.8 and 0.9, respectively. Units of *α* is RNA concentration/hr, where RNA concentration is defined by copies/voxel (voxel = 200 nm x 200 nm x 350 nm). Units of *β* is hr^−1^. **i**, Left: schematics of pulse-chase designs utilized in scEU-seq and scNT-seq; right: correlation of degradation between scEU-seq and scNT-seq after filtering genes based on fitting the degradation parameters using R^2^ > = 0.9. **j**, Histogram of estimated λ (nuclear export) values for all genes. **k**, The distribution of single cell-averaged DR values for all 991 genes across 0-6 hrs chase timepoints. n = 991 genes for each timepoint. Boxplots are defined in terms of mean (center line), 25-75% percentile (bounds of box), lower and upper quartile (whiskers). **l**, Histogram of estimated γ (cytoplasmic translocation) values for all genes. Blue dashed line separates the genes with γ > 0 and γ < 0, which indicates the opposite direction of observed translocation. **m**, Left, 19 genes with γ < 0 (R^2^ > 0.5) were strongly enriched in secreted and organellar proteins. Middle, time-lapsed DR values of representative genes. **n**, Schematics showing the observed inward direction of RNA translocation of genes with γ < 0. Two potential mechanisms are shown: 1). RNA translocation (active or passive); 2). Local RNA degradation.

To further quantify the kinetics during different stages of transcription and posttranscriptional processing, we estimated four key kinetic constants for all detected transcripts across RNA lifespan: synthesis (*α*), degradation (*β*), nuclear export (*λ*) (Fig. 3a) and cytoplasmic translocation (*γ*) (Fig. 3b). We noticed a correlation between physical cell volumes and number of single-cell RNA reads (Extended Data Fig. 4a,b). To correct for the potential bias caused by cell volume, we estimated *α* and *β* values based on the averaged concentrations of each RNA species (reads per voxel) across single cells (Extended Data Fig. 4c). Built on the previous studies^17,28^, our model assumed zero-order kinetics for *α* and first-order kinetics for *β*^29,30^. In addition, a threshold of fitting *β* for each gene (936 genes out of 991 genes with the coefficient of determination *R*^2^ ≥ 0.5) was applied for quality control purposes (Extended Data Fig. 4c–f). We further benchmarked our results against published single-cell sequencing datasets using EU labeling (scEU-seq^13^), which showed a moderate correlation of 0.30 (Pearson coefficient *r*) for *α* and *β* values (Extended Data Fig. 4g,h), which is comparable with two independent single-cell sequencing datasets (scEU-seq versus scNT-seq^10^, *r* = 0.39; Extended Data Fig. 4i). We attributed the variation across TEMPOmap, scEU-seq and scNT-seq to technical variations between imaging-based versus sequencing-based readout, different cell lines used in the studies and different pulse-chase experimental design. In parallel, we estimated the nuclear export rate (*λ*) based on the change in the ratios of nuclear-to-total reads over time. We noted that the estimation of *λ* might also be complicated by nuclear and cytoplasmic RNA degradation, and thus *λ* more accurately describes the change in the homeostasis of nucleocytoplasmic RNA distribution. Last, to systematically evaluate the relative positions of each RNA species in physical cytoplasmic space in 3D over time, we derived a distance ratio (DR)-based method (Extended Data Figs. 2d and 4c; Methods), where the cytoplasmic translocation rate (*γ*) was calculated by tracking the change of DR over time (Fig. 3b).

**Extended Data Fig. 5:**
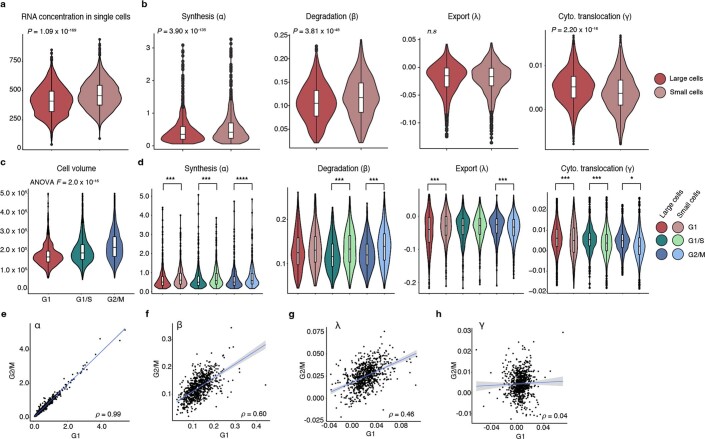
RNA kinetic parameter comparison by cell volume and cell-cycle stages. **a-b**, Violin plots showing the distribution of RNA concentration (**a**), synthesis, degradation, export and cytoplasmic translocation (**b**) for all detected genes averaged across single cells. n = 2792 cells for large cells and 2669 cells for small cells in **a-b**. **c**, violin plots showing the distribution of cell volumes (in voxels) in different cell-cycle stages. ANOVA test. n = 881, 2324, 2256 cells in G1, G1/S and G2/M, respectively. **d**, synthesis, degradation, export, and cytoplasmic translocation for all detected genes averaged across single cells in each cell cycle phase and separated by cell volumes. Units of *α* is RNA concentration/hr, where RNA concentration is defined by copies/voxel (voxel = 200 nm x 200 nm x 350 nm). Units of *β, λ, γ* are hr^−1^. Boxplots indicate mean and 25-75% quantile. n = 239, 1484, 1069 cells for G1, G1/S and G2/M of large cells, respectively, and 642, 840, 1187 cells for G1, G1/S and G2/M of small cells. Paired *t*-test. ***p < 0.001. *p < 0.05. **e-h**, Examples of pairwise correlation in Fig. 3d, showing scatter plots of the relationships between G1 and G2/M. Pearson’s correlation coefficients from left to right: *α* (*ρ* = 0.99), *β* (*ρ* = 0.60), *λ* (*ρ* = 0.46), *γ* (*ρ* = 0.04).

Notably, while nuclear export of RNA had been considered to be a constant in a previously published RNA velocity-based model ^30^, our analysis indicates that *λ* varies substantially among different RNA species, suggesting that homeostasis of nucleocytoplasmic transcript distribution is maintained by gene-specific regulatory mechanisms (Extended Data Fig. 4j). In addition, to our knowledge this is the first time it has been possible to systematically study the cytoplasmic translocation of RNAs of a large number of genes simultaneously at 1 h resolution. For most genes, *γ* > 0 (Extended Data Fig. 4k,l), implying transcript translocation from the nuclear membrane to the cytoplasmic membrane. However, we found a small subset of genes with *γ* < 0 (*R*^2^ > 0.5) that were significantly enriched in secreted and organellar proteins (Extended Data Fig. 4m), indicating possible relocation events from the cytosol to the endoplasmic reticulum (ER) or faster degradation rates for non-ER-anchored RNAs than ER-anchored ones. Further studies are required to investigate the kinetic mechanism that directs the cytoplasmic translocation of different RNA molecules (Extended Data Fig. 4n). Additionally, we observed that larger cells (as measured by cell volume) tend to exhibit slower synthesis (*α*) and degradation (*β*), but faster cytoplasmic translocation (*γ*) than small cells regardless of cell-cycle stages, whereas RNA export exhibits distinct trends in each cell-cycle phase (Extended Data Fig. 5a–d), indicating cell-size-dependent regulation of RNA kinetics.

Next, we asked whether any of the four RNA kinetic parameters were intrinsically coupled. Here, we performed pairwise correlations of the four parameters across 936 genes. We found that the overall correlation between each pair of parameters was weak (*ρ* < 0.1; Fig. 3c), suggesting that the kinetic parameters of RNA transcription, posttranscriptional processing^13^ and allocation are relatively independent^16^. We then explored the correlations of these kinetic parameters across the cell cycle. To this end, we performed a further pairwise correlation analysis of the four parameters across different genes at three cell-cycle phases (800 genes passed quality control; Fig. 3d; Methods). Interestingly, for each parameter, depending on its temporal sequence in the RNA life cycle, a trend of weakening correlations in cell-cycle phases emerged: at the early stage of RNA production, the synthesis rates *α* were highly correlated (*ρ* = 0.9–1.0; Extended Data Fig. 5e); during posttranscriptional processing in the nucleus, *λ* in the three phases have moderate correlations (*ρ* = 0.4–0.5; Extended Data Fig. 5g); near the end of the RNA life cycle, cytoplasmic translocation *γ* have much weaker correlations (*ρ* = 0–0.2; Extended Data Fig. 5h). This observation suggests that RNA metabolism and trafficking of different genes become less synchronized and increasingly heterogeneous from the upstream to the downstream stages of the RNA life cycle, potentially due to gene-specific and cell-cycle-dependent regulation.

**Extended Data Fig. 6:**
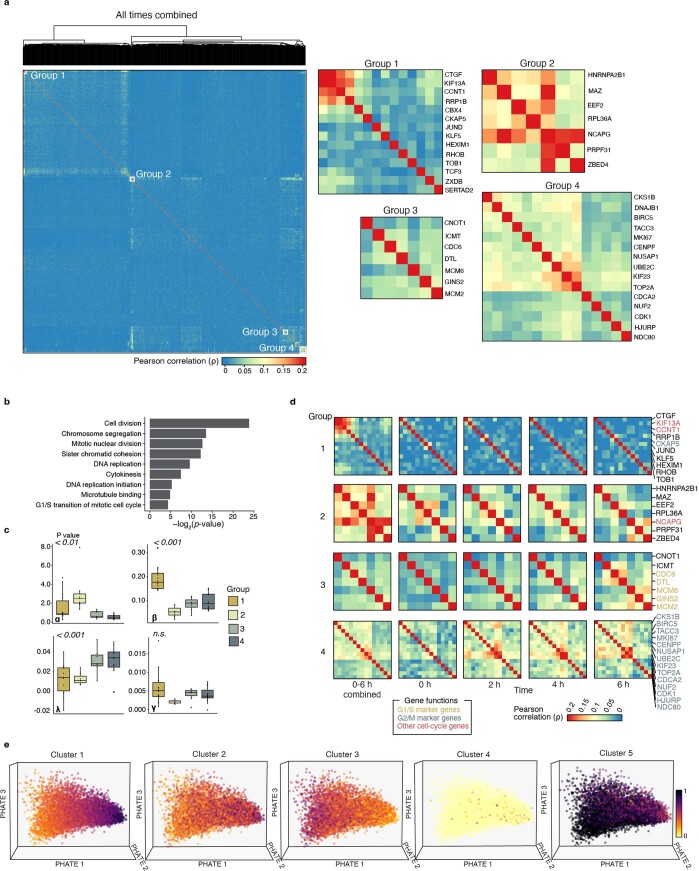
Single-cell RNA expression co-variation analysis. **a**, Heatmap depicting the pairwise correlation of 911 genes by TEMPOmap-measured single-cell RNA covariation when combining four timepoints (0, 2, 4, 6 hrs chase), where the color indicates the value of Pearson correlation. Group 1-4 are highlighted for highly correlated gene modules (left) and zoomed in (right). **b**, Pathway enrichment analysis result of genes in Group 1*-*4 from single-cell gene covariation heatmap in **a** using DAVID. **c**, Boxplots showing the distribution of six kinetic parameters of four gene groups (*n* = 14 genes in Group 1; *n* = 7 genes in Group 2, *n* = 7 genes in Group 3, *n* = 15 genes in Group 4). Units of *α* is RNA concentration/hr, where RNA concentration is defined by copies/voxel (voxel = 200 nm x 200 nm x 350 nm). Units of *β*, *λ*, *γ* are hr^−1^. *P* values, one-way ANOVA test. Data shown as means, 25-75% quartiles and ranges. **d**, Heatmaps were generated showing matrices of the pairwise gene co-variation *in each of the* 0, 2, 4 and 6 hrs chase timepoints. Gene order along each matrix was the same and determined by the hierarchical clustering tree of the matrix combining the four timepoints in **a** (results were not shown). Zoom-in views of Group 1-4 from the co-variation heatmaps generated by gene expression in each timepoint, showing the correlation of RNA co-variation of each gene module across individual timepoints. Color in heatmaps indicates the value of Pearson correlation. G1/S and G2/M marker genes were annotated in each gene block. **e**, PHATE visualization of single cells showing the expression level (the fraction of reads in the cluster/total reads) of Cluster 1-5.

Given the cell-cycle-resolved RNA kinetic landscape, we further investigated how RNAs could be dynamically ‘sculpted’ to fine-tune the temporal RNA expression profiles. First, we identified potentially coregulated RNAs through a pairwise single-cell covariation analysis of 936 genes from the aforementioned pulse-chase HeLa cell samples (1 h pulse, 0–6 h chase; Extended Data Fig. 6a, left). Using the matrix of pairwise correlation single-cell expression variation combining all timepoints (Methods), we identified four groups of genes with substantial intragroup correlation, indicating potential gene coregulation patterns (Group 1–4; Extended Data Fig. 6a, right). Notably, while these genes are enriched with cell-cycle-related functions (Extended Data Fig. 6b), the four groups differ significantly in several stages of RNA kinetics (Extended Data Fig. 6c). Next, we repeated the single-cell covariation analysis to each individual timepoint using the same gene order, and found that the shift in the covariation pattern of each group varies from 0 to 6 h (Extended Data Fig. 6d): Group 1 showed decreasing covariation pattern from 0 to 2 h postsynthesis; Group 2 showed consistently high expression covariation across time; in contrast, the covariation patterns of Group 3 and 4 emerged gradually from 2 h to 6 h postsynthesis. This observation suggests that, at the RNA level, cell-cycle progression is shaped jointly by an orchestration of genes with distinct transcriptional and posttranscriptional kinetic features.

### Differential RNA kinetic strategies by gene function

**Fig. 4:**
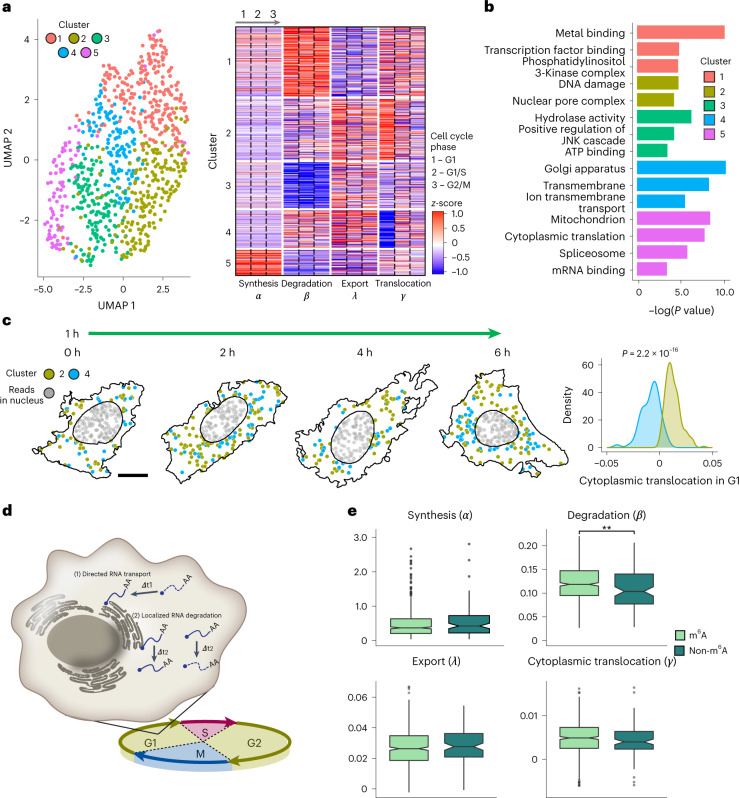
Differential RNA dynamics by gene function and posttranscriptional characteristics. **a**, UMAP representation (left) and heatmap (right) showing the gene clustering using all 12 estimated parameters across cell cycle. Color in the heatmap represents the parameter-wise *z*-score normalized value. **b**, Pathway enrichment analysis of genes in each cluster in **a** using DAVID. **c**, Left, visualization of cytoplasmic RNAs of Clusters 2 and 4 in representative cells across pulse-chase timepoints. Scale bar, 10 µm. Right, density plot showing the distributions of *γ* values of genes in clusters 2 and 4. **d**, Diagram illustrating two possible mechanisms of reverse mRNA translocation (*γ*) at the G1 phase of cluster 4 genes: directed RNA transport and localized RNA degradation. **e**, Boxplots comparing the four parameters estimated for m^6^A and non-m^6^A genes. Units of *α* is RNA concentration per hour, where RNA concentration is defined by copies per voxel (voxel = 200 nm × 200 nm × 350 nm). Units of *β*, *λ* and *γ* are per hour. *n* = 476 genes for m^6^A-modified and 89 genes for non-m^6^A-modified. Data shown as means (notches), 25–75% quartiles (boxes) and ranges (vertical lines). **P < 0.01, two-sided Wilcoxon test in **c** and **e**.

**Extended Data Fig. 7:**
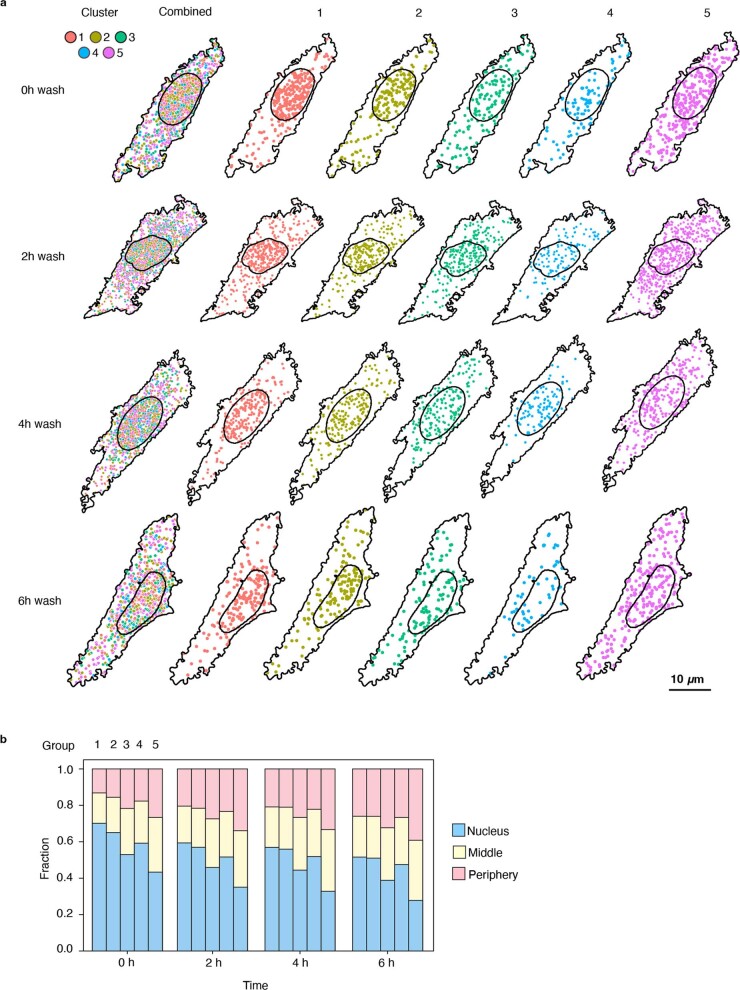
Visualization of gene clusters with different combinations of RNA kinetic strategies. **a**, Visualization of all the amplicons in representative cells (combined, left) and separated by gene clusters (right) across pulse-chase timepoints. Scale bar: 10 *µ*m. Colors of amplicons indicate unique gene clusters. **b**, Boxplots showing the subcellular distribution of RNA reads over time in each kinetic cluster. Vertical lines indicate s.d. For each cluster, 0, 2, 4, 6 hrs chase have *n* = 1028, 910, 946, 542 cells, respectively.

After recognizing the aforementioned four gene groups whose RNA temporal profiles coupled with cell-cycle phasing, we asked whether such correspondence between RNA kinetics and gene functions globally exists for other genes. To identify gene modules based on their shared kinetic patterns in the context of RNA life cycle and cell cycle, we first clustered 800 genes using the 12 parameters (four kinetic constants across three cell-cycle stages; Supplementary Table 2; Methods). The clustering analysis revealed five kinetic gene clusters of distinct kinetic landscapes (Fig. 4a) that also had distinct single-cell expressions (Extended Data Fig. 6e) and subcellular distributions over time (Extended Data Fig. 7a,b). Importantly, gene ontology (GO) analyses showed that the five clusters associate with distinct biological and molecular functions (Fig. 4b). For example, genes with unstable and slowly exported RNAs were strongly enriched in metal-binding and transcription factor binding activities (Cluster 1, *n* = 231 genes); genes with high RNA stability and moderate export rate (Cluster 3, *n* = 153 genes) were enriched in hydrolase and ATP-binding activities. In contrast, genes with rapid synthesis and greater RNA stability (Cluster 5, *n* = 86 genes) were enriched in constitutive cellular processes like mRNA splicing, translation and mitochondrial functions. We reasoned that these housekeeping genes tend to produce abundant and stable RNAs for a longer persistence of genetic information^28^.

**Extended Data Fig. 8:**
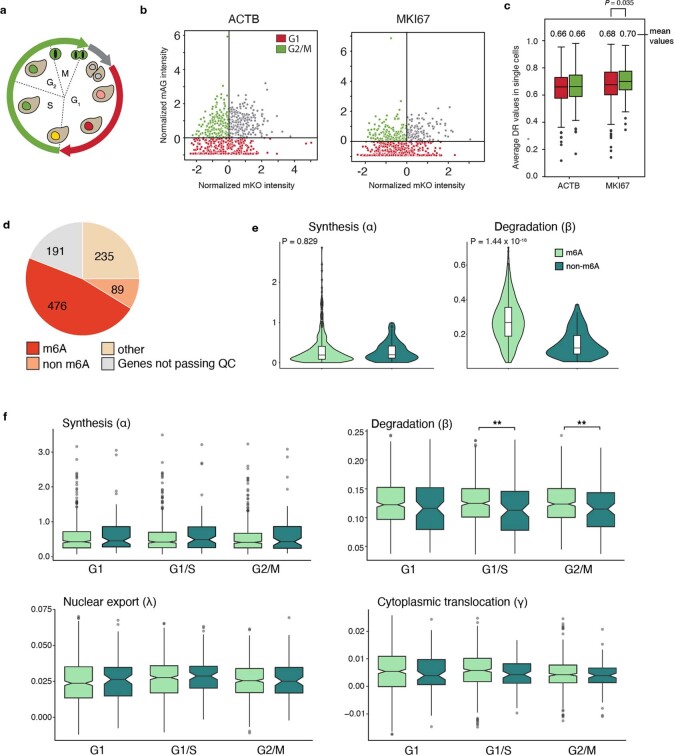
Validation of reverse translocation (*γ*) of cluster 4 genes in G1 by smFISH-HCR and subcellular RNA kinetics in the context of m^6^A post-transcriptional modification. **a**, schematics of HeLa-FUCCI cells. The cell line expresses two fluorescently-tagged cell cycle marker proteins (red, RFP-CDT1 and green, GFP-Geminin); these two markers have co-expression during G1-S transition (yellow). **b**, after plotting the normalized intensities of GFP and RFP, we obtained G1 and G2/M phases based on the thresholds indicated in the plots. **c**, boxplots comparing the DR values of ACTB and MKI67 in G1 and G2/M based on the cell label in **c**. **d**, Pie chart describing m^6^A-RNA methylation in the gene pool (see Methods). **e**, Violin plots showing the difference of synthesis (left) and degradation rates (right) between m^6^A-RNA and non-m^6^A-RNA from scEU-seq dataset. Paired t-test. Boxplots indicate mean and 25-75% quantile. **f**, Boxplots comparing the four parameters estimated for m^6^A (*n* = 476 genes) and non-m^6^A RNAs (*n* = 89 genes) across three cell-cycle phases. Units of *α* is RNA concentration/hr, where RNA concentration is defined by copies/voxel (voxel = 200 nm x 200 nm x 350 nm). Units of *β, λ, γ* are hr^−1^. ** *p* < 0.01, Wilcoxon test. Data shown as means (notches), 25-75% quartiles (boxes) and ranges (vertical lines).

Notably, while Cluster 2 (*n* = 205 genes) and 4 (*n* = 125 genes) are both characterized by slower synthesis, moderate degradation and faster export, they differ significantly in cytoplasmic translocation rates (*γ*), which are cell-cycle-dependent. In the G1 phase, RNAs of Cluster 2 exhibited higher *γ* than in other cell-cycle phases, whereas Cluster 4 showed the opposite trend (Fig. 4a, right). We found that Cluster 2 is enriched in DNA damage and repair genes while Cluster 4 genes are functionally related to organellar and membrane-bound proteins (Fig. 4b). Closer examination of the genes in Cluster 4 revealed that most (109 out of 125 genes) have negative *γ* values in G1, indicating an overall reverse direction of translocation in the G1 phase. Previous research has shown that many mRNAs encoding membrane-bound proteins were anchored to the surface of ER for localized protein synthesis^31^. We reasoned that the RNAs encoding these membrane proteins might also be regulated at the dynamic level, executed by both spatial and temporal localization control in a cell-cycle-dependent manner. Given that duplication of organelles and cell expansion are the major activities at G1 phase, our discovery suggests that ER-localized protein synthesis may be more active in G1, either by RNA transport towards the ER or local degradation of non-ER-anchored RNAs in the cell periphery (Fig. 4d and Extended Data Fig. 8a–c). Our proposed model provides a more comprehensive picture of the regulation dynamics of membrane protein at the RNA processing level from both spatial and temporal perspectives. Whereas the mechanism underlying our observed translocation data is still open to further investigation, these results highlight the importance of regulating the spatiotemporal localization of transcripts that carry different genetic information.

Next, we examined the RNA kinetic landscape in the context of *N*^6^-methyladenosine modifications (m^6^A)—a critical posttranscriptional chemical modification of RNA that plays vital physiological roles^32,33^. RNA methylation m^6^A is known to mediate a wide range of posttranscriptional gene regulation. However, the full landscape of the spatiotemporal dynamics of m^6^A-RNA has not been addressed systematically. To this end, we separated the genes encoding RNAs with and without m^6^A modifications by previous m^6^A profiling studies^34,35^ (m^6^A- or non-m^6^A-RNAs; Extended Data Fig. 8d; Methods). We observed that m^6^A-modified RNAs were significantly less stable than non-m^6^A-RNAs (higher *β*; Fig. 4e), consistent with an independently published dataset^13^ (Extended Data Fig. 8e). In addition, we observed the same trend when comparing the degradation constants in different cell-cycle phases, suggesting that regulation of m^6^A-methylated RNA decay is persistent throughout the cell cycle (Extended Data Fig. 8f). Together, these data demonstrate that TEMPOmap can be used to study spatiotemporal transcriptomics in combination with posttranscriptional modifications. TEMPOmap has the potential to facilitate multimodal transcriptomic analyses at single-cell and subcellular resolution.

### Applicability in human iPSC and primary cell cultures

**Fig. 5:**
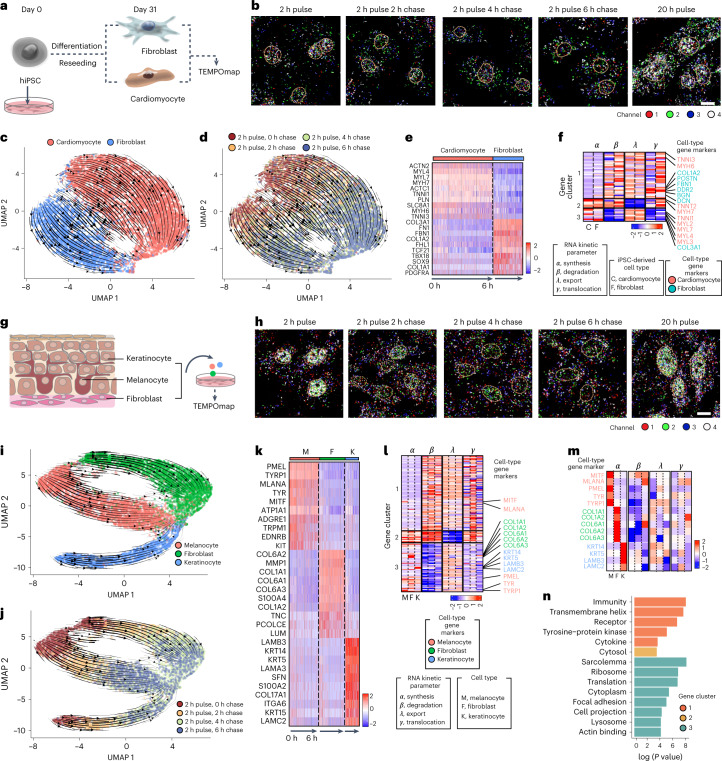
TEMPOmap reveals differential RNA kinetics in heterogeneous cell types. **a**, Schematics of hiPSC-CMs with mixed populations of CMs and cardiac fibroblasts. **b**, Representative fluorescent images of inprocess TEMPOmap with zoomed views of representative single cells of cycle 1 at each timepoint of hiPSC-CMs. Scale bar, 10 μm. **c**,**d**, Single-cell RNA UMAP embedding of nucleocytoplasmic matrix colored by identified cell types in hiPSC-CMs (**c**) and by labeling timepoints (**d**). **e**, Heatmap showing cell-type marker expression for each identified cell type (further separated into chase timepoints) in **c**. Color in the heatmap represents the *z*-score normalized value. **f**, heatmap showing the gene clustering using all eight estimated parameters in two cell types in hiPSC-CMs. Color in the heatmap represents the parameter-wise *z*-score normalized value. **g**, Schematics of isolation of primary human skin cells (melanocytes, keratinocytes and fibroblasts) from newborn foreskin tissues. **h**, Representative fluorescent images of inprocess TEMPOmap with zoomed views of representative single cells of cycle 1 at each timepoint of skin cell culture. Scale bar, 10 μm. **i**,**j**, single-cell RNA expression (measured by nucleocytoplasmic expression) visualized by UMAP and colored by identified cell types (**i**) and by labeling timepoints (**j**) in the skin cell dataset. **k**, Heatmap showing cell-type marker expression for each identified cell type (further separated by chase timepoints) in **i**. Color in the heatmap represents the *z*-score normalized value. **l**, Heatmap showing the gene clustering using all 12 estimated parameters in two cell types in skin cells. Color in the heatmap represents the parameter-wise *z*-score normalized value. **m**, Zoom-in heatmap of **l** showing the comparisons of *α*, *β*, *λ*, *γ* (from left to right) of cell-type marker genes in melanocytes, fibroblasts and keratinocytes. Marker genes for each of the three cell types are color-coded. **n**, Pathway enrichment analysis of genes in each cluster in **l** using DAVID.

**Extended Data Fig. 9:**
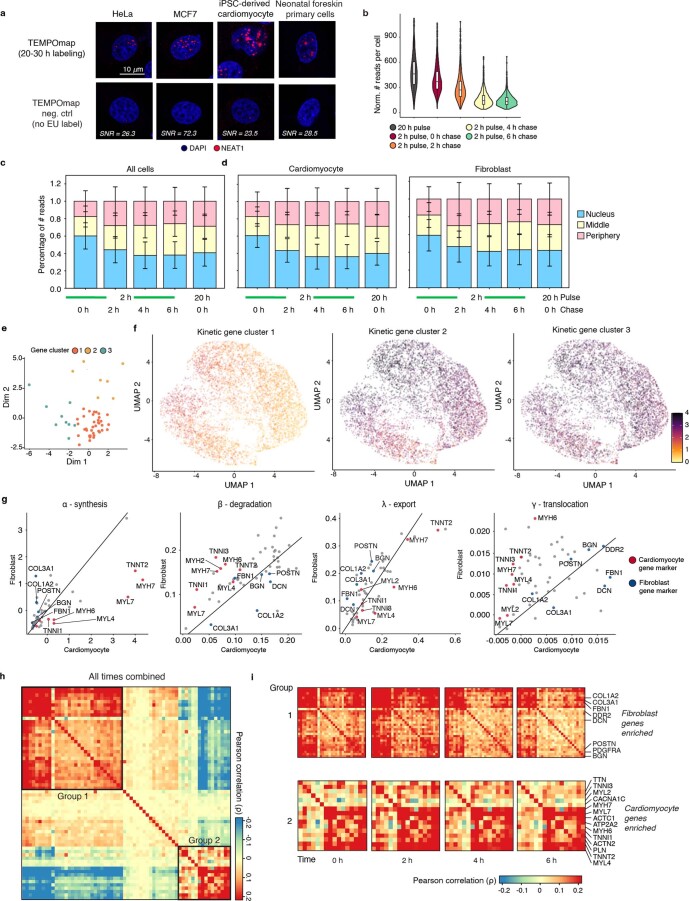
RNA subcellular dynamics in hiPSC-CMs. **a**, Representative fluorescent cell images of TEMPOmap detection of *NEAT1* in HeLa cells, MCF7, iPSC culture and primary foreskin cells. SNR (TEMPOmap 20-30 h labeling condition/No EU controls) in each cell line is shown in the images (n = 4-6 images). Scale bar: 10 μm. **b**, RNA reads (cDNA amplicons) per cell for each pulse-chase timepoint in hiPSC-CMs culture. **c-d**, stacked bar plots summarizing the fraction of reads in each subcellular region of all cells at each timepoint when combining all cells (**c**) and separating into CMs and cardiac fibroblasts (**d**). Vertical lines indicate s.d. **e**, PCA representation showing the gene clustering of 64 genes using all eight estimated parameters across cell cycle. **f**, UMAP visualization of single cells showing the averaged expression level of each gene in cluster 1-3 across all timepoints. Color indicates the level of averaged RNA expression. **g**, scatter plots showing the correlation of *α*, *β*, *λ*, *γ* (from left to right) in CMs and cardiac fibroblasts. Marker genes for CMs are colored in red, and for cardiac fibroblasts in blue. Units of *α* is RNA concentration/hr, where RNA concentration is defined by copies/voxel (voxel = 90 nm x 90 nm x 350 nm). Units of *β, λ, γ* are hr^−1^. **h-i**, Heatmap depicting the pairwise correlation of 64 genes in the hiPSC-CMs by TEMPOmap-measured single-cell RNA covariation when combining four chase timepoints (0, 2, 4, 6 hrs chase), where the color indicates the value of Pearson correlation (**h**). Group 1-2 are highlighted for highly correlated gene modules (left) and zoomed in (**i**).

**Extended Data Fig. 10:**
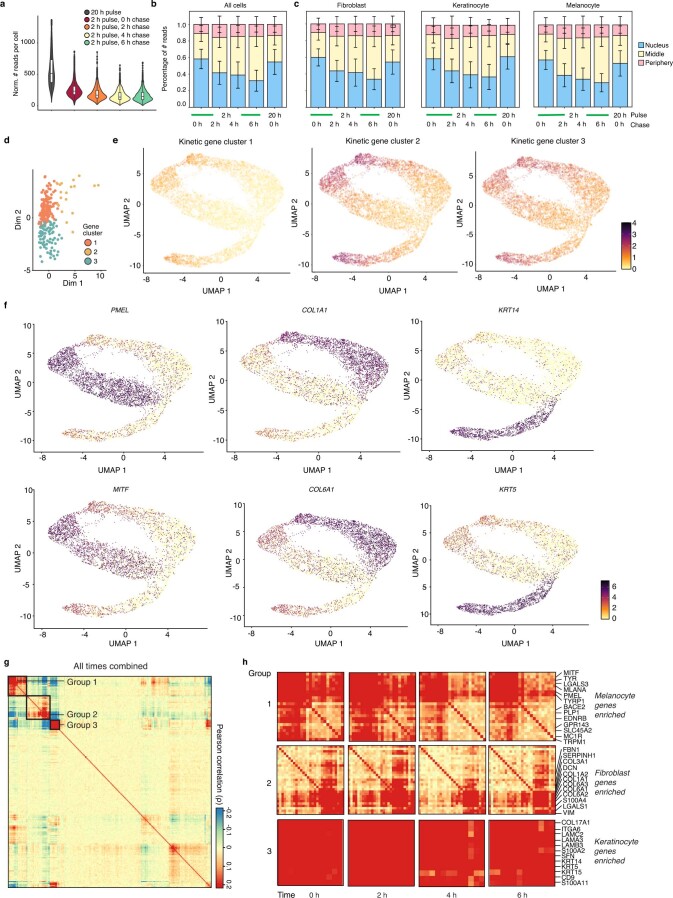
RNA subcellular dynamics in neonatal primary skin culture. **a**, RNA reads (cDNA amplicons) per cell for each pulse-chase timepoint in primary human skin cell culture. **b-c**, stacked bar plots summarizing the fraction of reads in each subcellular region of all cells at each timepoint when combining all cells (**b**) and separating into cardiomyocytes and fibroblasts (**c**). Vertical lines indicate s.d. **d**, PCA representation showing the gene clustering of 256 genes using all eight estimated parameters across cell cycle. **e**, UMAP visualization of single cells showing the averaged expression level of each gene in cluster 1-3 across all timepoints. Color indicates the level of averaged RNA expression. **f**, UMAP visualization of single cells based on the expression level of *PMEL* and *MITF* (melanocyte markers), *COL1A1* and *COL6A1* (fibroblast markers), and *KRT14* and *KRT5* (keratinocyte markers). Color indicates the level of RNA expression. **g-h**, Heatmap depicting the pairwise correlation of 256 genes in the primary skin cells by TEMPOmap-measured single-cell RNA covariation when combining four chase timepoints (0, 2, 4, 6 hrs chase), where the color indicates the value of Pearson correlation **(g)**. Group 1-3 are highlighted for highly correlated gene modules (left) and zoomed in **(h)**.

Finally, we investigated whether we could use TEMPOmap to study the spatiotemporal dynamics of mRNAs at different stages in heterogeneous cell types (Fig. 5 and Extended Data Figs. 9 and 10). To this end, we designed two sets of tri-probes and applied TEMPOmap to two different biological systems: human induced pluripotent stem cell-derived cardiomyocytes (hiPSC-CMs, mapping 64 genes; Fig. 5a) and primary human skin cells derived from neonatal foreskins (mapping 256 genes; Fig. 5g). For each of the cultures, we performed a pulse experiment using 5-EU for 2 h, followed by chase with 0, 2, 4 and 6 h. We then performed TEMPOmap cDNA amplicon library preparation, in situ sequencing, subcellular segmentation in 3D and data processing for both datasets (Extended Data Figs. 9b and 10a; Methods). As with the HeLa cells, we observed strong spatial relocation of RNAs in both hiPSC-CMs and primary skin cell cultures across the chase time (Fig. 5b,h).

All the cells (under 2 h pulse conditions) passed the quality control in hiPSC-CMs (*n* = 6,769 cells) and primary skin cell culture (*n* = 8,187 cells) were represented on the UMAP space based on single-cell RNA expression. Using the known marker genes for each cell type, we identified the populations of cardiomyocytes and cardiac fibroblasts in hiPSC-CMs culture, and melanocytes, keratinocytes and fibroblasts in skin cell culture as visualized on the UMAP plots (Fig. 5c,e,i,k). We then recapitulated the trajectories of single-cell transcriptomic profiles within each cell type across various chase times (Fig. 5d,j). Here, we observed a clear shift in RNA expression in the low-dimensional space from earlier to later timepoints for different cell populations when overlaid with streamlines indicating global transcriptomic shifts, which demonstrated that the temporally resolved RNA expression could also be distinguished in different cell types. In addition to the time-resolved transitions on the gene expression space, we could also observe physical transitions of the labeled transcripts in the three partitioned subcellular spaces (nuclei, middle and periphery) across time, when combining all the cells (Extended Data Figs. 9c and 10b) and when separating into individual cell types (Extended Data Figs. 9d and 10c).

To quantitatively evaluate the patterns of spatiotemporal RNA kinetics in all these cell types, we estimated synthesis (*α*), degradation (*β*), nuclear export (*λ*) and cytoplasmic translocation (*γ*) for all the genes passed the quality control in each cell type. Next, to further explore whether any of the kinetic parameters are cell-type dependent, we clustered 53 genes in the hiPSC-CMs dataset based on eight kinetic constants across two cell types (Extended Data Fig. 9e and Supplementary Table 3), and 245 genes in primary skin cell culture dataset based on 12 kinetic constants across the three cell types (Extended Data Fig. 10d and Supplementary Table 4). For the hiPSC-CMs dataset, we identified three kinetic gene groups (Fig. 5f), each with distinct combinations of kinetic regulation that contributed to varying RNA expression levels across chase time (Extended Data Fig. 9f). We also observed that cytoplasmic translocation (*γ*) generally has higher variations between CMs and cardiac fibroblasts than synthesis (*α*), degradation (*β*) and nuclear export (*λ*) (Fig. 5f), which may be related to the distinct cell morphologies of these two cell types. Interestingly, we also observed that cell-type markers are scattered throughout different kinetic gene clusters, yet they exhibit faster synthesis, faster nuclear export and slower degradation rates in their corresponding cell types than the other cell types (Extended Data Fig. 9g). Since cell-type marker genes are most correlated with specialized cell functions, this suggests that RNA kinetic regulation might prioritize the expression of most functionally important genes or facilitate the clearance of leaky transcripts from less functionally relevant genes (Extended Data Fig. 9h,i).

For the primary skin cell culture dataset, the kinetic clusters also showed consistent RNA dynamic patterns within each gene cluster across different cell types (Fig. 5l), suggesting that RNA kinetic parameters reflect intrinsic properties of each gene shared by different cell types. However, we also observed cell-type-specific synthesis and degradation rates of cell-type gene markers for melanocytes, fibroblasts and keratinocytes, which is consistent with our observations in cell-type markers of iPSC dataset (Fig. 5m and Extended Data Fig. 10e,f). GO analysis of each kinetic gene cluster revealed that transcripts with high degradation and low nuclear export rate are enriched in cytosolic proteins; genes with moderate degradation and cytoplasmic translocation are enriched in transmembrane proteins and immunity; genes with lower degradation and moderate translocation are enriched in the ribosome and cell structures (Fig. 5n). These results suggest that cells use distinct combinations of multistep kinetics for functionally distinct genes, which shapes the RNA expression levels of the gene clusters across time (Extended Data Fig. 10g,h). Overall, our result in primary cell cultures echoed the previous observations in HeLa cells that RNAs of different molecular and physiological functions are dynamically ‘sculpted’ across their lifetime.

## Discussion

TEMPOmap is a new in situ transcriptomic platform that simultaneously profiles time- and space-resolved transcriptomics in single cells, a multimodal single-cell transcriptomics technology at a subcellular resolution that has not been achieved before. We demonstrated its ability to systematically detect the subcellular allocation and cytoplasmic translocation of transcripts over time. More importantly, our study provided a full landscape of RNA subcellular kinetics at the single-cell level and revealed how RNA kinetics contribute to cellular functions such as cell-cycle progression. We observed a strong correlation of RNA kinetic patterns and the molecular functions of genes, suggesting that this function-oriented regulation of RNA life cycle may have evolved to control spatiotemporal gene expression in a precise and economic way^28^. We also demonstrated the broad applicability of TEMPOmap to human iPSC-derived cell culture and primary cell culture, revealing cell-type-dependent regulation of RNA kinetics. From our analyses of HeLa cells, hiPSC-CMs and primary skin cell cultures, we observed that RNA kinetics generally reflect intrinsic features for each gene grouped by their molecular functions. However, the kinetics parameters of genes that are key to specialized cellular functions are strongly dependent on cell state and cell type, emphasizing the importance of studying subcellular RNA kinetics at the single-cell level. It is noteworthy that the detection efficiency of TEMPOmap may have sequence biases (for example, U-rich sequence), since it requires the metabolic labeling of U analogs and relies on DNA probe design. In future work, TEMPOmap can be combined with high-throughput single-cell functional genomics (for example, CRISPR screens^36^) to determine key molecular factors that impact the kinetic landscape of RNA life cycle. With optimization of metabolic labeling conditions^15,37,38^ and integration of various molecular probing schemes, this methodology can be adapted for ex vivo or in vivo tissue samples to systematically profile dynamic events in tissue biology. Furthermore, such spatiotemporally coordinated transcriptomic patterning may shed light on the molecular mechanisms of various biological phenomena, including development and pattern formation, learning and memory, biological clocks, and disease pathogenesis and progression.

## Methods

### Chemicals and enzymes

Chemicals and enzymes: Gel Slick Solution (Lonza, catalog no. 50640); PlusOne Bind-Silane (GE Healthcare, catalog no. 17-1330-01); poly-d-lysine solution, 50 μg ml^–1^ (ThermoFisher, catalog no. A3890401); ultrapure distilled water (Invitrogen, catalog no. 10977-015); glass bottom 24-well plates (Greiner Bio-One, catalog no. 662892, and MatTek, catalog no. P24G-1.5-13-F); no. 2 Micro coverglass, 12 mm diameter (Electron Microscope Sciences, catalog no. 72226-01); 16% paraformaldehyde (PFA), EM grade (Electron Microscope Sciences, catalog no. 15710-S); methanol for high-performance liquid chromatography (Sigma-Aldrich, catalog no. 34860-1L-R); PBS, 7.4 (Gibco, catalog no. 10010-023 for 1× and catalog no. 70011-044 for 10×); Tween-20, 10% solution (Calbiochem, catalog no. 655206); Triton X-100, 10% solution (Sigma-Aldrich, catalog no. 93443); OminiPur Formamide (Calbiochem, catalog no. 75-12-7); 20× SSC buffer (Sigma-Aldrich, catalog no. S6639); ribonucleoside vanadyl complex (New England Biolabs, catalog no. S1402S); yeast tRNA (Invitrogen, catalog no. AM7119); SUPERase·In (Invitrogen, catalog no. AM2696); 5-EU (Invitrogen, catalog no. E10345); 1.5× Click buffer (Lumiprobe, catalog no. 61150); l-ascorbic acid (Sigma-Aldrich, catalog no. A5960); T4 DNA ligase, 5 Weiss U μl^–1^ (Thermo Scientific, catalog no. EL0011); Phi29 DNA polymerase (Thermo Scientific, catalog no. EP0094); 10 mM dNTP mix (Invitrogen, catalog no. 100004893); BSA, molecular biology grade (New England Biolabs, catalog no. B9000S); 5-(3-aminoallyl)-dUTP (Invitrogen, catalog no. AM8439); BSPEG9 (Thermo Scientific, catalog no. 21582); methacrylic acid NHS ester, 98% (Sigma-Aldrich, catalog no. 730300); dimethylsulfoxide, anhydrous (Molecular Probes, catalog no. D12345); acrylamide solution, 40% (Bio-Rad, catalog no. 161-0140); Bis Solution, 2% (Bio-Rad, catalog no. 161-0142); ammonium persulfate (Sigma-Aldrich, catalog no. A3678); N,N,N′,N′-tetramethylethylenediamine (Sigma-Aldrich, catalog no. T9281); OminiPur SDS, 20% (Calbiochem, catalog no. 7991); Antarctica Phosphatase (New England Biolabs, catalog no. M0289S); 4,6-diamidino-2-phenylindole (DAPI) (Molecular Probes, catalog no. D1306); Flamingo Fluorescent Protein Gel Stain (Bio-Rad, catalog no. 1610491); DMEM medium (ThermoFisher, catalog no. 11995); FBS (HyClone, catalog no. SH3007103); Lipofectamine RNAiMAX (Invitrogen, catalog no. 13778075); azidobutyric acid NHS ester (Lumiprobe, catalog no. 63720); Bio-Spin P-6 Columns, SSC buffer (Bio-Rad, catalog no. 7326002); Human Melanocyte Medium (Gibco, catalog no. M254CF500); Human Melanocyte Growth Supplement (Gibco, catalog no. S-002-5); Y-27632 (Sigma, catalog no. Y0503); HCR RNA-FISH Buffers and Amplifiers (Molecular Instruments)^39^.

### Design and construction of TEMPOmap probes

TEMPOmap tri-probes were designed to contain a set of three separate DNA oligonucleotide probes: splint, primer and padlock. DNA splint was prepared by incubating 40 µM 5′ amino-modified splint oligo (manufactured by Integrated DNA Technologies (IDT)) with 25 mM azidobutyric acid NHS ester (azide-NHS) in 0.1 M NaHCO_3_ at room temperature overnight. The product was purified by ethanol precipitation and run through Bio-Spin P-6 Columns (SSC buffer).

A representative sequence graph is shown in Extended data Fig. 1i. The probes were designed as follows: (1) The 5′ azide-modified splint is divided into two regions: a linker containing 50 adenosine nucleotides connected to a 12-nucleotide splint-padlock annealing sequence. To be protected from enzymatic amplification, the splint contains a 3′ terminal inverted dT and the phosphorothioate bonds on the last three nucleotides at the 3′-end of the oligo. The splint-padlock annealing sequence enables the hybridization of the splint with padlock on the same RNA, creating a double-strand DNA region with a ‘nick’ that can be sealed in the ligation step. (2) The 5′ phosphorylated padlock is comprised of the complementary splint-padlock annealing sequence, two regions of the same 5-nucleotide barcode, a 10-nucleotide primer-padlock annealing sequence, a 19–25 nucleotide target region for specific RNA binding and several short linkers. (3) The primer contains another 19–25 nucleotide target region, 2-nucleotide mismatch bases, a 5-nucleotide linker and a 5-nucleotide gene-unique sequence that is reverse complementary to the barcode on the matching padlock. The two target regions in each set of the primer and the padlock reside one to two bases next to each other on the same mRNA species. Splint, primer and padlock probes targeting ACTB mRNA are included in Supplementary Table 5-1.

The detailed procedure of target region selection on primer and padlock was applied as previously described^1^. In brief, we only considered the shortest isoforms and the coding regions except for noncoding RNAs. Picky v.2.2^40^ was used to design the target sequence on each probe pair with the length range of 40–46 nucleotides and six sequences were selected for each gene. The cDNA sequences of the selected regions were split into halves of 20–25 nucleotides separated by 0–2 nucleotides, which contained the best match of melting temperature. The probes were pooled, ordered and manufactured by IDT. The reading and decoding probes used in SEDAL sequencing were designed and ordered according to Wang et al.^1^.

For constructing TEMPOmap bi-probes (splint and padlock), the design of the splint probe was the same as described in the tri-probe section. Each padlock probe contains a 40-nucleotide target region selected as described in the tri-probe design and five sequences were selected for each gene.

### TEMPOmap gene selection

For 1000-gene in HeLa cells, we selected the transcripts based on the following criteria: (1) the expression of each transcript is above 20 reads per kilobase per million reads mapped from a few RNA-seq data^33,34,41^; (2) transcripts that have diverse subcellular locations^16^; (3) transcripts that mark specific cell-cycle phases^25^ and (4) transcripts that are known to be m^6^A-modified and m^6^A-devoid. We did not consider the length of transcripts as our selection criteria.

For 64-gene in hiPSC-CM culture and 256-gene in primary skin culture, we selected transcripts based on cell-type gene markers as well as other genes that we found to be cell-type-specific and interesting in the biological system from the previous RNA-seq reports (skin 256-gene list: Joost et al.^42^; Ji et al.^43^; Tirosh et al.^25^; Jerby-Arnon et al.^44^. hiPSC-CMs 64-gene list: Friedman et al.^45^; Cui et al.^46^; Lee et al.^47^; Biendarra-Tiegs et al.^48^; Zhao et al.^49^).

### HeLa cell lines and culture conditions

The human HeLa cell line used in this study was purchased from ATCC (CCL-2) and grown in DMEM (Gibco, catalog no. 11995) medium supplemented with 10% FBS. The cells were plated on 24-well pretreated glass bottom plates (treatment described in next section) and grown at 37 °C with 5% CO_2_ before the TEMPOmap experiment.

### hiPSC culture and differentiation

hiPSCs were purchased from the WiCell research institute. Authentication and testing for the mycoplasma were conducted by WiCell. hiPSC cells were maintained on Matrigel-coated plates at 37 °C and 5% CO_2_ with Essential 8 medium (Gibco). hiPSC-CMs were generated based on methods described previously with minor modification^44,45^. Briefly, hiPSCs were cultured in a six-well plate with E8 medium for 3–4 days to 70–80% confluency. Next, the E8 medium was removed. RPMI 1640 medium plus 1% B27-insulin and 12 μM CHIR99021 was added for cardiac differentiation (day 0). On day 1, the medium was changed to RPMI 1640 medium plus 1% B27-insulin. On day 3, the medium was changed to RPMI 1640 medium plus 1% B27-insulin and 5 μM IWR1. On day 5, the medium was changed to RPMI 1640 medium plus 1% B27-insulin. On day 7, the medium was changed to RPMI 1640 medium plus 1% B27. The medium was then replaced with fresh RPMI 1640 medium plus 1% B27 every other day. The hiPSC-CMs started beating from day 8 or day 10. Then, hiPSC-CMs (day 29 of differentiation) were reseeded on the Magrigel-coated 24-well plate in RPMI 1640 medium plus 1% B27 and rock inhibitor (RI, 5 μM), and left for another 2 days for TEMPOmap data collection; 1 mM EU was used to metabolically label the cells. All experiments involving human cells were approved by the Harvard University IRB and ESCRO committees (IRB20-0249).

### Isolation and culture of primary human skin cells

Isolation and culture of primary human skin cells followed previously published protocols^50,51^. Briefly, newborn foreskin tissues were incubated with 2.5 mg ml^–1^ Dispase solution at 4 °C overnight. The following day, the epidermis and dermis were separated from each other with fine forceps. For isolation of keratinocytes (epidermal cells) and melanocytes, the epidermal tissues were incubated with 0.05% trypsin for 15–30 min at 37 °C, then neutralized with 10% FBS in DMEM, filtered (100 μm filter, Millipore), centrifuged and rinsed with 10% FBS-DMEM to obtain cell pellets that contained both keratinocytes and melanocytes. The cell pellets were resuspended and seeded with the human melanocyte medium plus human melanocyte growth supplement and 10 mM Y-27632. For isolation of dermal cells, dermal tissues were chopped and incubated with type I collagenase (2.5 mg ml^–1^) at 37 °C for 1 h, then the digestion was neutralized with 10% FBS in DMEM. The digested cell solutions were filtered, centrifuged and washed with 10% FBS-DMEM to obtain dermal cell pellets. The dermal cell pellets were resuspended and seeded with 10% FBS-DMEM. The medium was changed every 2 days for primary cell culture until cells reached confluency.

Cells isolated from epidermal tissue, containing both keratinocytes and melanocytes, were combined with dermal cells isolated from dermal tissue at a 4:1 ratio, and a total of 0.2 × 10^6^ mixed cells were seeded into each well of a 24-well plate, and cultured with the above human melanocyte medium plus 10% DMEM containing 10% FBS. After 2 days, the cells were prepared for TEMPOmap data collection; 1 mM EU was used to metabolically label the cells.

The procedure for obtaining newborn foreskin tissues from discarded hospital specimens without any personal identity information was approved by the Partners Human Research Committee/IRB (protocol 2013P000093).

### TEMPOmap experimental procedure

The 24-well glass bottom plates were treated with 1% methacryloxypropyltrimethoxysilane (Bind-Silane) and poly-d-lysine solution sequentially before cell plating. For iPSC culture, the plates were additionally coated with Matrigel (dilution, 1:80 in DMEM/F12 medium) at 37 °C for 2 days. Cells were then plated on the coated plates and maintained in growth medium (DMEM containing 10% FBS) in a humid culture incubator with 5% CO_2_ at 37 °C. Pulse-chase experiments were performed with 200 μM 5-EU and washed with cell medium for a designated amount of time. After metabolic labeling and washing, the cells were fixed with 1.6% PFA in PBS for 10 min and permeabilized with prechilled (−20 °C) methanol for 30 min at −80 °C. The samples were then taken from –80 °C, equilibrated to room temperature and quenched with buffer containing PBSTR (0.1% Tween-20, 0.1 U μl^–1^ SUPERase•In in PBS) supplemented with 10 mM Tris pH 7.5 and 0.1 mg ml^–1^ yeast tRNA for 10 min.

To functionalize the nascently ethynylated RNAs, 5′ azide-modified DNA splint (5 µM) was added to 1× Lumiprobe click chemistry buffer supplemented with 500 μM dNTP. CuAAC was initiated by adding ascorbate (800 µM). The reaction mixture was incubated at 37 °C for 1 h with gentle shaking. The samples were then washed with PBSTR at 37 °C for 10 min twice.

A library of TEMPOmap primer and padlock probes (targeting 991 genes) and a set of STARmap SNAIL probes (targeting METTL3/14, YTHDF1-3) were separately pooled and ordered from IDT. All of the probe pools were dissolved in ultrapure RNase-free water to 100 nM per oligonucleotide for storage. The probe mixtures were then heated at 90 °C for 5 min and cooled on ice. Subsequently, the samples were incubated in 1× hybridization buffer (2× SSC, 10% formamide, 1% Tween-20, 20 mM RVC, 0.1 mg ml^–1^ yeast tRNA, 0.2 U μl^–1^ SUPERase•In) supplemented with TEMPOmap probes at 2 nM per oligo and STARmap probes at 10 nM per oligo in a 40 °C humidified oven with gentle shaking for 14–16 h. The samples were then washed with PBSTR twice and high salt buffer (4× SSC in PBSTR) once at 37 °C for 20 min in each wash, and one more PBSTR rinse after the wash. The samples were then incubated with T4 DNA ligation mixture (1:20 dilution of T4 DNA ligase, 1× BSA and 0.2 U μl^–1^ SUPERase•In) at room temperature for 2 h with gentle shaking, followed by washing twice with PBSTR. Subsequently, the samples were incubated with RCA mixture (1:20 dilution of Phi29 DNA polymerase, 250 µM dNTP, 20 µM 5-(3-aminoallyl)-dUTP, 0.2 U µl^–1^ SUPERase•In, 1× BSA) at 30 °C for 2 h with gentle shaking, followed by PBST (0.1% Tween-20 in PBS) wash twice. Next, the samples were treated with 25 mM Methylacrylic acid NHS ester (MA-NHS) in 0.1 M NaHCO_3_ at room temperature for 2 h, followed by washing once in PBST.

To cast the gel, the samples were first incubated with monomer buffer (4% acrylamide, 0.2% bis-acrylamide, 2× SSC) supplemented with 0.2% TEMED at room temperature for 15 min. The buffer was removed and 30 µl polymerization mixture (0.2% ammonium sulfate, 0.2% TEMED dissolved in monomer buffer) was added slowly to the center of the sample, which was immediately covered with Gel Slick-coated coverslip. The sandwiched polymerization mixture was incubated for 1 h in N_2_ followed by two washes in PBST. The gelated samples were then treated with dephosphorylation mixture (1:100× dilution of Antarctic phosphatase, 1× BSA) at room temperature overnight followed by washing twice with PBST.

### TEMPOmap detection on dual luciferase reporter transcripts

The HeLa Tet-OFF cell line was used to control inducible gene expression by adding/removing doxycycline (DOX). Luciferase reporter plasmid containing firefly luciferase (expressed after inducible promoter) was provided by X. Wang^34^. Next, the reporter plasmid was transfected into HeLa Tet-OFF cell lines and the cells were incubated with 1 μg ml^–1^ DOX during passaging and maintenance. For Extended Data Fig. 1h, the two EU labeling points were incubated with 200 μM EU and no DOX for 1 h and 20 h, respectively. For Extended Data Fig. 3b, a pulse-chase experiment with DOX withdrawal was designed as illustrated in the figure. For all timepoints, cells were incubated with 1 μg ml^–1^ DOX to keep firefly luciferase expression off, while it was turned on by removing DOX while EU was washed off. Next, TEMPOmap on firefly luciferase and STARmap on renilla luciferase (for transfection control and gene expression normalization) were performed in the same cells. For both firefly and renilla luciferases, six sets of probes were designed for amplicon library preparation.

### Total EU-RNA detection by fluorescently labeled streptavidin

After the pulse-chase labeling experiment in HeLa cells described in the previous section and in Fig. 2a, cells were fixed and permeabilized and the total EU-RNAs were biotinylated with 10 μM azide-PEG9-biotin via a CuAAC-mediated click reaction. The biotin-modified RNAs were incubated with streptavidin-AF647 overnight at 4 °C. The fluorescent intensity of streptavidin-AF647 was measured to estimate EU-RNA localization across time. Subcellular segmentation was performed by CellProfiler.

### TEMPOmap sample imaging and in situ sequencing

TEMPOmap imaging and in situ sequencing were carried out as previously described^1^ with the following modifications. In brief, we performed six rounds of four-color confocal imaging for 998 gene measurements, plus one final round including the nucleus detection stained with DAPI, cell morphology stained with flamingo and the ER region with Concanavalin A following the manufacturers’ instructions. Each round of imaging began by incubating the samples with the sequencing mixture (1:25 dilution of T4 DNA ligase, 1× BSA, 10 µM reading probe and 5 µM fluorescent decoding oligonucleotides) at room temperature for 3 h, followed by rinsing with washing and imaging buffer (2× SSC, 10% formamide) three times for 5 min each before imaging. After image acquisition, the samples were treated with the stripping buffer (60% formamide, 0.1% Triton X-100) twice for 10 min followed by washing three times with PBST. After six rounds of imaging, we then imaged the cell nucleus stained with DAPI, cytoplasm stained with flamingo fluorescent gel stain and ER stained with Concanavalin A. Images were acquired using Leica SP8 confocal microscopy with a 405 diode, white light laser, ×40 oil-immersed objective (numerical aperture 1.3). For each round, images were acquired with Alexa 488, 546, 594 and 647 illumination at 30 focal planes. For HeLa data collection, the voxel size of the imaging was 200 nm × 200 nm × 350 nm. For iPSC and skin data collection, the voxel size of the imaging was 90 nm × 90 nm × 350 nm.

### Image processing and amplicon decoding

First, an image deconvolution was applied with Huygens Essential v.20.10.1p2. The deconvolved images were then normalized by the Min-Max strategy and further adjusted with histogram equalization where images in the first sequencing round were used as reference. In addition, a customized Top-hat filtering was applied to enhance fluorescence signals. To better identify the barcode of each cDNA amplicon, both a global registration and a nonrigid registration were performed on the preprocessed images. Global image registration was accomplished using a 3D fast Fourier transform (FFT) to compute the cross-correlation between two image volumes at all translational offsets. The position of the maximal correlation coefficient was identified and used to translate image volumes to compensate for the offset. The nonrigid registration was achieved by the ‘imregdemons’ function in MATLAB v.2020b. After registration, individual amplicons were identified in each color channel on the first round of sequencing. For this experiment, amplicon dots were identified by finding local maxima in 3D with MATLAB function ‘imregionalmax’. Dots with intensity at their centroids less than the threshold were removed. Based on the estimation of amplicon size, the dominant color for each dot across all four channels on each round was determined by a three by three by three voxel volume surrounding the dot centroid. The integrated intensity of the voxel volume in each channel was used for color determination. In this case, each dot in each round had an L2-normalized vector with four elements. The color of each dot was determined by the corresponding channel with the highest value in the vector. Dots with several maximum values in the vector were discarded. Then, dots were first filtered based on quality scores (average of –log(color vector value in dominant channel) across all sequencing rounds). The quality score quantified the extent to which each dot on each sequencing round came from one color rather than a mixture of colors. The barcode codebook was converted into color space based on the expected color sequence following the two-base encoding of the barcode DNA sequence. Dots passed the quality threshold and with a matched barcode sequence in the codebook were kept; all other dots were rejected. Both the physical locations and gene identities of the filtered dots were saved for downstream analysis.

### Cell segmentation and subcellular segmentation

Image segmentation was performed using CellProfiler v.4.1.3 and other customized scripts in MATLAB v.R2020b. A two-dimensional reference segmentation mask was generated by a customized pipeline for both the DAPI staining image and the composite image combining amplicon channels and flamingo fluorescent gel staining image.

For 3D segmentation, the images targeting different cellular compartments were first processed by a median filter and binarized with the Otsu’s method. All connected components (objects) that have fewer than 100 pixels were removed from the binary image. Then, images were dilated with a disk structure element with radius equal to three. Lastly, a 3D segmentation mask targeting each cellular region was generated by an element-wise multiplication process between the binary image and the two-dimensional reference cell segmentation from the previous step. The 3D volume of each cell was calculated by the total voxels of the single cell in 3D segmentation. The nucleus regions were removed from the 3D cell segmentation masks to create the cytoplasm segmentation.

Filtered amplicons overlapping each segmented cell region in 3D were then assigned to the specific subcellular region (Extended Data Fig. 2b), to compute a per-cell gene expression matrix in each cellular compartment.

### RNA subcellular distribution analysis via DR calculation

To quantify the relative location of reads inside the cytoplasm, a DR was calculated for each of the cytoplasmic reads. The DR value for an RNA read within a cell was defined as the shortest distance of the read to the surface of nucleus (*d*_n_), defined by nucleus segmentation, normalized by the sum of this distance and the shortest distance to surface of the cell membrane (*d*_c_), defined by cell segmentation (Extended Data Fig. 2d). The shortest distance was calculated with a Euclidean distance transform function provided in Scipy. A cutoff DR value of 0.909 (or *d*_n_/*d*_c_ = 10) was used to further segment the cytoplasmic region into ‘middle’ and ‘periphery’ for a detailed examination of the subcellular distribution RNA reads across timepoints in TEMPOmap dataset.

### Dynamic modeling of RNA cytoplasmic translocation

RNA cytoplasmic translocation parameter *γ* was estimated with a linear regression on mean DR value (described above) for each gene across different pulse-chase timepoints (Fig. 3b). For each gene, the DR values of all the reads in all the corresponding cells were averaged for the mean DR value to represent its cytoplasmic localization at a particular timepoint. Linear regression was performed across all timepoints for each gene using the ‘linregress’ function of Scipy in Python.

### Cell clustering visualization via PHATE based on single-cell and subcellular-resolved gene expression matrix

Single-cell clustering was performed on the cell-by-gene expression matrix, normalized to a same number of cell total reads. Subcellular-resolved clustering was performed on a horizontally concatenated nuclear and cytoplasmic expression matrix, both of identical dimension as the cell-by-gene expression matrix, and normalized by the method described above. For both matrices, PHATE was used as the clustering and visualization method, which has shown to preserve both local and global structure of the data. A neighbor parameter of 30 in PHATE was used in both analyses.

### RNA degradation kinetics vector visualization and transcriptomic vector field animation by dynamo

The quivers (arrows overlaid with dots) on (I, III; Fig. 2e) were constructed by projecting the transcriptomic dynamics considering total RNA degradation kinetics in single cells to the PHATE embeddings. The total RNA degradation rates for vector visualization were estimated by – degradation rate × *n*, where *n* is the nascent RNA. Only a subset of quivers is visualized to avoid crowding of quivers. Quivers are sampled based on the inverse of local density of each cell to ensure relative uniform coverage across the entire space, Note we do not explicitly model the total RNA velocity because we do not capture the RNA synthesis with our pulse-chase labeling strategy.

The transcriptome vector field animation was constructed from the same RNA degradation kinetics as described above using dynamo^22^. Basically, we first learned the continuous vector fields in 3D PHATE space with dynamo. Then, a subset of cells at timepoint 0 were sampled whose vector field trajectories from these initial points were predicted by numerically integrating from those points with the function of the reconstructed vector field. We then animated the predicted movement in the PHATE space of those cells over time.

### Cell-cycle phase classification and validation

The three cell-cycle phases of single cells (G1, G1/S, G2/M) were classified using the TEMPOmap nascent RNA expression of the following genes via the cell-cycle scoring function ‘score_genes_cell_cycle’ in scanpy.

G1/S genes: *BCL2L1*, *CDC6*, *DSCC1*, *DTL*, *MCM5*, *UNG*, *SNN*, *FEN1*, *GINS2*, *GMNN*, *MCM2*, *MCM4*, *MCM6*, *PCNA*, *PRIM1*, *RRM1*, *TYMS*, *UHRF1*, *CDCA7*.

G2/M genes: *TOP2A*, *TPX2*, *UBE2C*, *HJURP*, *BIRC5*, *CCNB2*, *CDCA2*, *CKAP5*, *CKS1B*, *CKS2*, *HMGB2*, *NCAPD2*, *NDC80*, *NUF2*, *TACC3*, *TMPO*, *MKI67*, *CENPF*.

To validate whether 1 h-labeled nascent transcriptome can accurately assign cell-cycle phases, we repeated the cell-cycle scoring analysis using 1 h pulse and total transcripts (22 h pulse and 0 h chase) from the previously published scEU-seq dataset, and conducted a correlation analysis of the assigned cell-cycle results (Extended Data Fig. 3d).

### Dynamic modeling of RNA nuclear export

RNA nuclear export parameter *λ* was estimated with a linear regression fit to the ratios of nuclear read fraction across timepoints via the following equation (Extended Data Fig. 4c):

$${{y}} = - \lambda \times t + {{{\mathrm{constant,}}}}$$

where

$$\begin{array}{*{20}{c}} {y = {{{\mathrm{nuclear}}}}\,{{{\mathrm{reads}}}}/\left( {{{{\mathrm{nuclear}}}} + {{{\mathrm{cytoplasmic}}}}\,{{{\mathrm{reads}}}}} \right), } \\ {\lambda = {{{\mathrm{nuclear}}}}\,{{{\mathrm{export, and}}}}} \ {t = {{{\mathrm{time}}}}\,{{{\mathrm{point.}}}}} \end{array}$$

We made an assumption of constant *λ* over time. For each gene, *y* was calculated by the averaged nuclear reads per averaged single-cell reads across all cells at a particular timepoint. Linear regression was performed across all timepoints for each gene using the 'lm' function in R.

### Dynamic modeling and fitting of RNA synthesis and degradation

#### Calculating RNA concentrations

After obtaining RNA copy numbers of genes in nucleus and cytoplasm of single cells, we first normalized the reads across different chase timepoints against the averaged reads of control genes (that is, genes targeted by STARmap probes. For HeLa dataset: METTL3, METTL14, YTHDC2, YTHDF1-3; for hiPSC-CM and primary skin cells: HPRT1), which we assumed to display uniform expression under different pulse-chase conditions since the total RNAs of each gene were targeted. We then divided normalized RNA copy numbers (Extended Data Fig. 4a) by unit cell volume (in voxels) to calculate the RNA concentrations in single cell (*X*(*t*)). RNA concentrations (copies per voxel) have a unit in reads per voxel and will be denoted as [RNA] in the following section. Voxel = 200 nm × 200 nm × 350 nm in HeLa and 90 nm × 90 nm × 350 nm in iPSC and skin cells.

#### Modeling

Let *α* be the transcription constant ([RNA] h^–1^) and *β* be the degradation constant (1 h^–1^). The time derivative of *X*(*t*) at 1 h pulse is described by a first-degree ordinary differential equation, assuming parameters *α* and *β* are all constants (similarly for all other parameters in other equations below):

1 $${\mathrm{d}}X(t)/{\mathrm{d}}t = \alpha - \beta \times X(t)$$

And the time derivative of *X(t)* during the subsequent chase timepoints is described by:

2 $${\mathrm{d}}X(t)/{\mathrm{d}}t = - \beta \times X(t)$$

Note that we assumed there is no new RNA synthesized after 1 h pulse. Using equations (1) and (2), we estimated *α* and *β* from single-cell RNA concentration for each gene. It should also be noted that we found the data at 1 h pulse to 1 h chase condition to be an outlier of our linear model, potentially because of residual EU in the cells after washing. Therefore, we removed the cells from 1 h chase and used only the cells from 0, 2, 4 and 6 h chase for parameter estimations.

#### Fitting and thresholding

We evaluated the goodness of the fitting of our model to the data using *R*^2^. We further restricted to genes that (1) exhibit positive values; (2) have *R*^2^ ≥ 0.5 when fitting Eq. (2) to estimate *β*. We also assumed constant degradation (*β*) coefficient in RNA concentration over time. After evaluating the genes that passed the fitting threshold, we obtained 972 genes with all four parameters (*α*, *β*, *λ*, *γ*) when we combined all cells, and 800 genes for all 12 parameters (four parameters across three cell-cycle phases) when we separated cells into three cell-cycle phases.

#### Validation of kinetic parameter estimation

To validate our models, we repeated the calculation of synthesis (*α*) and degradation (*β*) using RNA copy numbers per cell of TEMPOmap dataset and the published scEU-seq dataset (1 h pulse, 0, 2, 4 and 6 h chase), where we obtained 417 overlapping genes.

It is noteworthy that previous studies reported that RNA synthesis rate is higher in G2/M phase than in G1 (ref. ^52^). When repeating the calculation of *α* and *β* using RNA copy numbers per cell from our TEMPOmap data and published scEU-seq data^13^, we observed consistent results that RNA synthesis rate is higher by around 15% in G2/M, whereas degradation rates across the cell cycle follow different trends between the two datasets, potentially because of different cell lines. However, when estimating *α* and *β* using RNA concentrations (RNA copy numbers per unit nuclei volume for *α* or that per unit cell volume for *β*), we observed no substantial changes in the distribution of *α* and *β* values at different cell-cycle stages. The contrast of estimated *α* and *β* by RNA copy number per cell versus RNA concentrations agrees with previous works that showed transcription rate is proportional to available sites of the chromosomes and gene expression homeostasis are regulated by cell size. The method details of gene clustering and visualization are described in the next section.

### Kinetic parameter correlation and clustering analyses

Matrices describing pairwise correlation coefficients of the estimated kinetic parameters were constructed for both four parameters that are cell-cycle-combined (consisting of 972 genes) and 12 parameters that are cell-cycle-resolved (consisting of 800 genes) using R, which were then visualized by scatterplot matrix (Fig. 3c) and heatmap (Fig. 3d), respectively. Representative examples of the correlations of cell-cycle-resolved 12 parameters were also visualized as scatterplots in Extended Data Fig. 5e-h.

We next performed dimensionality reduction on the cell-cycle-resolved 12 kinetic parameters of 800 genes using UMAP and then gene clustering by Louvain, via repurposing code implemented in Seurat v.3 at 1.0 resolution, which resulted in five gene clusters. We noted that the variance across kinetic parameters varies more greatly than the variance of the same parameter in different cell-cycle phases. Therefore, to better visualize the kinetic differences among the four clusters, we computed *z*-scores for each kinetic parameter calculated of all genes among the three cell-cycle phases and plotted the *z*-scores as heatmap in Fig. 4a.

For each of the five clusters identified by UMAP analysis, GO was performed on the genes in each of the four clusters identified by UMAP analysis against TEMPOmap 991-gene list as the background using DAVID^53,54^. All the GO terms with statistical significance (*P* value is close to or below 0.05) are shown in Fig. 4b. Visualization of the five clusters in representative cells was performed using customized scripts in Python.

### Covariation analysis of single-cell nascent RNA expression over time

For the covariation analysis in single-cell RNA expression across time (Extended Data Fig. 6a,d), we first normalized single-cell RNA reads according to the procedure when constructing PHATE embedding (see above), and constructed a matrix describing the Pearson correlation of expression level between each gene pair using all the cells from 0, 2, 4 and 6 h chase. We then conducted a hierarchical clustering to organize the genes based on their correlation coefficients in these time-combined cells using ComplexHeatmap package embedded functions. The matrix was then visualized by heatmap (Extended Data Fig. 6a, left). We then created heatmaps describing the correlation coefficients of expression level in each individual timepoint while retaining the same grouping and ordering of the genes in all of the matrices (Extended Data Fig. 6d, complete heatmaps not shown). Comparing the heatmaps across the four timepoints, we manually identified four small gene clusters (annotated as group 1–4 in Extended Data Fig. 6a) that showed correlated expressions when combining all the timepoints but also varied along individual timepoints (Extended Data Fig. 6d). GO analysis was performed on all the genes in four groups against the background gene list as described above using DAVID^53,54^.

### Definition of m^6^A genes in TEMPOmap gene list

For m^6^A-related gene labels, genes encoding high-confidence m^6^A-modified transcripts were identified by previously reported photoactivatable ribonucleoside-enhanced crosslinking and immunoprecipitation (PAR-CLIP) and immunoprecipitation data. Briefly, we defined m^6^A-RNA as (1) having an enrichment at least onefold in nonfragmented m^6^A RIP-seq and (2) transcripts that were bound in each replicate of PAR-CLIP. Similarly, we defined non m^6^A-RNA as (1) having an enrichment of less than zero in nonfragmented m^6^A RIP-seq and (2) transcripts that have no peak in either replicate of PAR-CLIP. Using these criteria, we defined 573 genes encoding m^6^A-RNA and 111 genes encoding non m^6^A-RNA from the TEMPOmap gene list.

### Statistics and reproducibility

The representative cell images in Fig. 2f (V) were from one TEMPOmap experiment consisting of 2,256 G2/M cells. The representative cell images in Fig. 5b,h were from two TEMPOmap experiments consisting of 6,769 cells and 8,187 cells, respectively.

### Reporting summary

Further information on research design is available in the Nature Portfolio Reporting Summary linked to this article.

## Online content

Any methods, additional references, Nature Portfolio reporting summaries, source data, extended data, supplementary information, acknowledgements, peer review information; details of author contributions and competing interests; and statements of data and code availability are available at 10.1038/s41592-023-01829-8.

## Supplementary information

Supplementary Information Supplementary Note.

Reporting Summary

Supplementary Table The workbook includes Supplementary Tables 1–5. Table 5 is further separated into Table 5-1, 5-2, 5-3 and 5-4.

## Data Availability

TEMPOmap sequencing datasets of 991-gene in HeLa cells, 64-gene in hiPSC-CMs and 256-gene in skin cells are available in the Single Cell Portal (https://singlecell.broadinstitute.org/single_cell/study/SCP1792) and Zenodo (10.5281/zenodo.7623400). The kinetic parameters of HeLa cells, hiPSC-CMs and skin cells are available in the Supplementary Tables. scEU-seq data were accessed under GSE128365. scNT-seq data were accessed under GSE141851. Bulk RNA expression data were accessed from Wang et al. (10.1038/nature12730)^34^.

## Code availability

TEMPOmap is implemented based on MATLAB v.R2020b, Python v.3.8 and R v.3.6.3. The following packages and software were used in data analysis: CellProfiler v.4.1.3^55^, ImageJ v.1.51, anndata v.0.7.5, matplotlib v.3.1.3, seaborn v.0.11.0, scanpy v.1.8.2^56^, numpy v.1.19.4, scipy v.1.6.3, pandas v.1.3.5, scikit-learn v.1.0.2, numba v.0.54.1, tifffile v.2021.7.2, scikit-image v.0.18.3, Seurat v.3.2.2^57^, SeuratDisk v.0.0.0.9013, ggplot2 v.3.3.5, factoextra v.1.0.7, ComplexHeatmap v.2.7.10.9001, dplyr v.1.0.4, circlize v.0.4.13 and IRanges v.2.20. The TEMPOmap analysis tool will be maintained and updated at https://github.com/wanglab-broad/TEMPOmap. RNA vector field is generated by Dynamo v.1.0.0 and is available at https://github.com/aristoteleo/dynamo-release.
