# Supplementary material for: Spatiotemporally resolved transcriptomics reveals the subcellular RNA kinetic landscape
Source: Nat Methods. 2023 Apr 10;20(5):695–705. doi: 10.1038/s41592-023-01829-8 (PMC10172111; doi:10.1038/s41592-023-01829-8)
Supplement: Supplementary file 1 — Supplementary Note. [file 41592_2023_1829_MOESM1_ESM.pdf]

nature methods

Article

https://doi.org/10.1038/s41592-023-01829-8

Spatiotemporally resolved transcriptomics reveals the subcellular RNA kinetic landscape

In the format provided by the authors and unedited

Supplementary Notes

Difference in kinetic parameters estimated by TEMPOmap and other studies

The differences in the estimated mRNA synthesis and degradation rates from different datasets could be caused by (**1**) different cell lines, culture conditions, and species; (**2**) intrinsically different experimental procedures for DNA library preparation; (**3**) differences in data processing and normalization (as also brought up in comment 2).

(**1**) TEMPOmap, scEU-seq, and scNT-seq utilized different cell lines (**Supplementary Note Table 1**), which may have different RNA kinetics across cell types and species.

(**2**) Since the RNA synthesis is estimated as zero-order kinetics in TEMPOmap and other studies, the absolute RNA synthesis rate for each gene is proportional to the capture/detection efficiency of EU-labled RNA. scEU-seq utilized straptavidin-modified beads to capture biotinylated RNAs, which were subsequently converted to double-strand DNA, fragmented, and tagged for next-generation sequencing. In contrast, TEMPOmap utilized *in situ* hybridization-based approach to target RNAs of interest, and the probes were subsequently amplified into "rolonies" and detected via sequencing-by-ligation technology. Thus, the fundamental difference between the library preparation protocols and data processing could be contributing to the recovery of RNA readout, which directly influenced the estimation of kinetic parameters. We think the higher synthesis rate estimated by TEMPOmap is due to higher detection efficiency for imaging-based approach (skipping capturing/purification steps), analogous to smFISH (close to 100% efficiency given optimized probe design) versus scRNA-seq (5-20%).

(**3**) In contrast, the RNA degradation rates are estimated by first-order kinetics, which is based on the relative changes among different time points in each dataset and influenced by data analysis and normalization approaches. As we discussed above, the intrinsic experimental difference between sequencing-based (scEU-seq and scNT-seq) and imaging-based (TEMPOmap) methods should entail different data normalization strategies (**Supplementary Note Table 1**). We would also like to point out that the sequencing-based reactions give relative abundance measurements of labeled RNA (i.e. labeled RNAs/(labeled + unlabeled RNAs)), whereas TEMPOmap did not measure unlabeled RNAs. Here, our normalization strategy for TEMPOmap was to use STARmap-targeting genes (six genes in 1000-gene HeLa data, see "Dynamic modeling and fitting of RNA synthesis (α) and degradation (β)" under the Method section). We think the observed differences may reflect differences in experimental procedures (4SU or 5EU, sequencing or imaging) and data processing (read normalization). However, as we demonstrated in our response to comment 2, the STARmap normalization strategy should be a valid approach. The apparent slower degradation kinetics observed in TEMPOmap versus scEU-seq and scNT-seq may due to different technical biases or different dynamic ranges among the three methods: sequencing-based approaches may have nonlinear drop of detection efficiency for low abundant RNAs (underestimation of RNAs at later chase time points, thus faster degradation kinetics) whereas imaging-based approach may reach optical saturation for high abundant RNAs (multiple amplicons of the same gene may be optically resolved as one amplicon, which may lead to underestimation of RNAs at earlier time points and thus slower degradation kinetics).

Supplementary Note Table 1 | Comparison of cell lines and normalization strategies for kinetic estimation among scEU-seq, scNT-seq and TEMPOmap.

Method	Cell line	Gene count normalization strategy for kinetic estimation
scEU-seq	Human FUCCI-RPE1	mean sum of total UMI $\times \left(\frac{mean\ labeled\ UMI\ for\ gene\ in\ pool\ of\ cells}{sum\ of\ total\ UMI\ for\ pool\ of\ cells\ of\ the\ time\ point}\right)$
scNT-seq	Mouse mESC	The fraction of labeled transcripts was calculated with summed labeled UMI counts divided by total UMI counts (labeled and unlabeled). The fractions of labeled transcripts were corrected for doubling time (14.8 h) and normalized to 0 h of chase.
TEMPOmap	Human HeLa cells	Mean amplicons for STARmap genes in single cells of 0h chase $\times \left(\frac{\text{Mean amplicon counts for TEMPOmap genes (labeled transcripts)in single cells}}{\text{Mean amplicons for STARmap genes in single cells of the time point}}\right)$
