# Supplementary material for: Spatiotemporally resolved transcriptomics reveals the subcellular RNA kinetic landscape
Source: Nat Methods. 2023 Apr 10;20(5):695–705. doi: 10.1038/s41592-023-01829-8 (PMC10172111; doi:10.1038/s41592-023-01829-8)
Supplement: Supplementary file 2 — Reporting Summary [file 41592_2023_1829_MOESM2_ESM.pdf]

nature portfolio

Corresponding author(s): Xiao Wang

Last updated by author(s): Feb 9, 2023

Reporting Summary

Nature Portfolio wishes to improve the reproducibility of the work that we publish. This form provides structure for consistency and transparency in reporting. For further information on Nature Portfolio policies, see our Editorial Policies and the Editorial Policy Checklist.

Statistics

For all statistical analyses, confirm that the following items are present in the figure legend, table legend, main text, or Methods section.

n/a	Confirmed	
☐	☒	The exact sample size (*n*) for each experimental group/condition, given as a discrete number and unit of measurement
☐	☒	A statement on whether measurements were taken from distinct samples or whether the same sample was measured repeatedly
☐	☒	The statistical test(s) used AND whether they are one- or two-sided<br>*Only common tests should be described solely by name; describe more complex techniques in the Methods section.*
☒	☐	A description of all covariates tested
☐	☒	A description of any assumptions or corrections, such as tests of normality and adjustment for multiple comparisons
☐	☒	A full description of the statistical parameters including central tendency (e.g. means) or other basic estimates (e.g. regression coefficient) AND variation (e.g. standard deviation) or associated estimates of uncertainty (e.g. confidence intervals)
☐	☒	For null hypothesis testing, the test statistic (e.g. *F*, *t*, *r*) with confidence intervals, effect sizes, degrees of freedom and *P* value noted<br>*Give P values as exact values whenever suitable.*
☒	☐	For Bayesian analysis, information on the choice of priors and Markov chain Monte Carlo settings
☒	☐	For hierarchical and complex designs, identification of the appropriate level for tests and full reporting of outcomes
☐	☒	Estimates of effect sizes (e.g. Cohen's *d*, Pearson's *r*), indicating how they were calculated

Our web collection on statistics for biologists contains articles on many of the points above.

Software and code

Policy information about availability of computer code

Data collection	Leica LAS-X microscope imaging software 3.5.5.19976 was used during data acquisition. Huygens 20.10 was used for image deconvolution.
Data analysis	MATLAB R2020b was used for imaging analysis of in situ sequencing images. Python 3.8 and R 3.6.3 were used as a platform for some of the downstream analysis. The following packages and software were used in data analysis: CellProfiler 4.2.4, ImageJ 1.51, anndata 0.7.5, matplotlib 3.1.3, seaborn 0.11.0, scanpy 1.8.2, numpy 1.19.4, scipy 1.6.3, pandas 1.3.5, scikit-learn 1.0.2, numba 0.54.1, tifffile 2021.7.2, scikit-image 0.18.3, Seurat 3.2.2, SeuratDisk 0.0.0.9013, ggplot2 3.3.5, factoextra 1.0.7, ComplexHeatmap 2.7.10.9001, dplyr 1.0.4, circlize 0.4.13, IRanges 2.20. RNA vector field is generated by Dynamo 1.0.0 (https://github.com/aristoteleo/dynamo-release). The TEMPOmap codes will be available and maintained at https://github.com/wanglab-broad/TEMPOmap.

For manuscripts utilizing custom algorithms or software that are central to the research but not yet described in published literature, software must be made available to editors and reviewers. We strongly encourage code deposition in a community repository (e.g. GitHub). See the Nature Portfolio guidelines for submitting code & software for further information.

Data

Policy information about availability of data

All manuscripts must include a data availability statement. This statement should provide the following information, where applicable:

- Accession codes, unique identifiers, or web links for publicly available datasets
- A description of any restrictions on data availability
- For clinical datasets or third party data, please ensure that the statement adheres to our policy

TEMPOmap sequencing datasets of 991-gene in HeLa cells, 64-gene in hiPSC-CMs and 256-gene in skin cells are available in the Single Cell Portal (https://singlecell.broadinstitute.org/single_cell/study/SCP1792) and Zenodo (https://doi.org/10.5281/zenodo.7623400). The kinetic parameters of HeLa cells, hiPSC-CMs

and skin cells are available in the Supplementary Table. scEU-seq data were accessed under GSE128365. scNT-seq data were accessed under GSE141851. Bulk RNA expression data were accessed from Wang et al (https://doi.org/10.1038/nature12730).

Field-specific reporting

Please select the one below that is the best fit for your research. If you are not sure, read the appropriate sections before making your selection.

☒ Life sciences ☐ Behavioural & social sciences ☐ Ecological, evolutionary & environmental sciences

For a reference copy of the document with all sections, see nature.com/documents/nr-reporting-summary-flat.pdf

Life sciences study design

All studies must disclose on these points even when the disclosure is negative.

Sample size	Experiments were applied on 18,176 HeLa cells, 6,769 hiPSC-CM cells, 8,187 primary skin cells. The sample size was determined based on the previous scEU-seq dataset, which described at least 15 cells per cell cycle per time point were used to determine the parameters. We include ~600 cells per time point per cell cycle (type) for the kinetic estimation.
Data exclusions	During the single cell transcriptomic analysis, cells with reads less than 300 reads were removed to exclude potential cell residues. For hiPSC-CMs, cells bigger than 3x 10^6 voxels are removed to prevent doublets. For skin cells, cells with reads less than 40 reads were removed to exclude potential cell residues.
Replication	Major findings in TEMPOmap dataset were drawn from one replicate (n = 18,176 cells for HeLa cells, n = 6,769 cells for hiPSC-CM, n = 8,187 cells for skin cells).
Randomization	Randomization is not applicable to this study because the different pulse-chase time points of TEMPOmap were known throughout the experiments, data acquisition and analysis.
Blinding	Blinding is not applicable to this study because the different pulse-chase time points of TEMPOmap were known throughout the experiments, data acquisition and analysis.

Reporting for specific materials, systems and methods

We require information from authors about some types of materials, experimental systems and methods used in many studies. Here, indicate whether each material, system or method listed is relevant to your study. If you are not sure if a list item applies to your research, read the appropriate section before selecting a response.

Materials & experimental systems

n/a	Involved in the study	
☒	☐	Antibodies
☐	☒	Eukaryotic cell lines
☒	☐	Palaeontology and archaeology
☒	☐	Animals and other organisms
☒	☐	Human research participants
☒	☐	Clinical data
☒	☐	Dual use research of concern

Methods

n/a	Involved in the study	
☒	☐	ChIP-seq
☒	☐	Flow cytometry
☒	☐	MRI-based neuroimaging

Eukaryotic cell lines

Policy information about cell lines

Cell line source(s)	1. Human HeLa cell line: purchased from ATCC (CCL-2, lot 70016358).<br>2. Human FUCCI cell line: purchased from RIKEN BRC.<br>3. Human induced pluripotent stem cells (hiPSC, hiPSC-IMR90-1): WiCell Research Institute (Madison, WI, USA).<br>4. Human primary skin cells: The procedure for obtaining newborn foreskin tissues from discarded hospital specimens without any personal identity information was approved by the Partners Human Research Committee/IRB (protocol 2013P000093).
Authentication	1. The cell line has been authenticated by the STR method.<br>2. The cell line has been authenticated by the STR method.<br>3. Authentication were performed by WiCell Research Institute.
Mycoplasma contamination	1. The cell lines were confirmed as mycoplasma negative by DAPI (4",6-diamidino-2-phenylindole) or Hoechst DNA staining and microscope imaging.

2. Test for the mycoplasma contamination were performed by RIKEN BRC.
3. Test for the mycoplasma contamination were performed by WiCell Research Institute. We also confirmed that the cell line was negative for mycoplasma contamination before all experiments.

Commonly misidentified lines (See ICLAC register)	None.
